# Routine cardiac biomarkers for the prediction of incident major adverse cardiac events in patients with glomerulonephritis: a real-world analysis using a global federated database

**DOI:** 10.1186/s12882-024-03667-y

**Published:** 2024-07-22

**Authors:** Elin Mitford Davies, Benjamin J. R. Buckley, Philip Austin, Gregory Y. H. Lip, Louise Oni, Garry McDowell, Anirudh Rao

**Affiliations:** 1 Department of Women’s and Children’s Health, Institute of Life Course and Medical Sciences, University of Liverpool, Liverpool, UK; 2 Department of Nephrology, Liverpool University Hospitals NHS Foundation Trust, Liverpool, UK; 3 Liverpool Centre for Cardiovascular Science at University of Liverpool, Liverpool John Moores University and Liverpool Heart and Chest Hospital, Liverpool, UK; 4 Cardiovascular Health Sciences, Research Institute for Sport and Exercise Sciences, Liverpool John Moores University, Liverpool, England, UK; 5 TriNetX Inc, London, England; 6 Danish Centre for Health Services Research, Department of Clinical Medicine, Aalborg University, Aalborg, Denmark; 7 Department of Paediatric Nephrology, Alder Hey Children’s NHS Foundation Trust Hospital, Eaton Road, Liverpool, UK; 8 School of Pharmacy and Biomolecular Sciences, Liverpool John Moores University, Liverpool, England, UK; 9 Institute of Life Course and Medical Sciences, University of Liverpool, Liverpool, England, UK; 10 Research Laboratory, Liverpool Heart and Chest Hospital, Liverpool, England, UK

**Keywords:** Glomerulonephritis, Biomarker, Cardiovascular, MACE, Mortality

## Abstract

**Rationale & objective:**

Glomerulonephritis (GN) is a leading cause of chronic kidney disease (CKD). Major adverse cardiovascular events (MACE) are prolific in CKD. The risk of MACE in GN cohorts is multifactorial. We investigated the prognostic significance of routine cardiac biomarkers, Troponin I and N-terminal pro-BNP (NT-proBNP) in predicting MACE within 5 years of GN diagnosis.

**Study Design:**

Retrospective cohort study.

**Setting & participants:**

Data were obtained from TriNetX, a global federated health research network of electronic health records (EHR).

**Exposure or predictor:**

Biomarker thresholds: Troponin I: 18 ng/L, NT-proBNP: 400 pg/mL.

**Outcomes:**

Primary outcome: Incidence of major adverse cardiovascular events (MACE). Secondary outcome: was the risk for each individual component of the composite outcome.

**Analytical Approach:**

1:1 propensity score matching using logistic regression. Cox proportional hazard models were used to assess the association of cardiac biomarkers with the primary and secondary outcomes, reported as Hazard Ratio (HR) and 95% confidence intervals (CI). Survival analysis was performed which estimates the probability of an outcome over a 5-year follow-up from the index event.

**Results:**

Following PSM, 34,974 and 18,218 patients were analysed in the Troponin I and NTproBNP cohorts, respectively. In the Troponin I all cause GN cohort, 3,222 (9%) developed composite MACE outcome HR 1.79; (95% CI, 1.70, 1.88, *p* < 0.0001). In the NTproBNP GN cohort, 1,686 (9%) developed composite MACE outcome HR 1.99; (95% CI, 1.86, 2.14, *p* < 0.0001).

**Limitations:**

The data are derived from EHR for administrative purposes; therefore, there is the potential for data errors or missing data.

**Conclusions:**

In GN, routinely available cardiac biomarkers can predict incident MACE. The results suggest the clinical need for cardiovascular and mortality risk profiling in glomerular disease using a combination of clinical and laboratory variables.

**Supplementary Information:**

The online version contains supplementary material available at 10.1186/s12882-024-03667-y.

## Introduction

Chronic Kidney disease (CKD) is a global health economic burden and contributes to premature mortality. In 2017, CKD was ranked as the 12th leading cause of death, with Cardiovascular Disease (CVD) deaths attributed to CKD representing 4·6% of total mortality [1]. CKD is a chronic systemic pro-inflammatory state contributing to vascular and myocardial remodelling, atherosclerosis, vascular calcification and complex dyslipidaemia [2, 3]. Importantly, CKD is an independent risk factor for CVD [4], with the risk of cardiovascular (CV) events more clinically significant than the development of kidney failure in those with CKD [5].

Glomerulonephritis (GN) is one of the leading causes of CKD [6]. Patients with GN have a higher absolute risk of developing CVD [7]. The risk of CVD in GN is multifactorial, including exposure to immunosuppressive medication which can increase likelihood of developing CVD [8]. Furthermore, there is emerging evidence of the pro-inflammatory consequences of GN and the development of a unique cardiovascular phenotype [9]. Following diagnosis, patients with GN may initially have a stable level of renal function alongside significant proteinuria, an independent risk factor for CVD [10].

Given the multifactorial relationship between GN and the development of CV complications, patients diagnosed with GN must be appropriately monitored for their risk of CVD. The study aimed to investigate the prognostic significance of routinely measured circulating plasma cardiac biomarkers such as Troponin I or N-terminal pro-BNP (NT-proBNP) in predicting major adverse cardiovascular events (MACE) within 5 years of diagnosis of GN in a global federated research network database (TriNetX).

## Methods

### Study Design

A retrospective cohort study was based on anonymised data from TriNetX, a global federated health research network that provides anonymised access to electronic health records (EHR). The TriNetX database of longitudinal data includes demographics with laboratory and mortality data derived from the EHR of large healthcare organisations (HCOs). The dataset represents the Global Collaborative Network of 113 healthcare organisations of > 140 million patients, primarily in North America and Western Europe. The diagnosis has been standardised to the International Statistical Classification of Diseases and Related Health Problems 10th Revision, Clinical Modification (ICD-10CM) [11], allowing the accurate identification of disease cohorts. More information on TriNetX can be found online (https://trinetx.com/about-trinetx/). The data used in this analysis were accessed on 10th March 2024.

### Building cohorts in TriNetX

All patients with a diagnosis of a Primary GN (as coded by ICD-10CM: N00-N08 in their EHR); IgA nephropathy (IgAN); membranous nephropathy (MN); focal segmental glomerulosclerosis (FSGS); or minimal change disease (MCD) were included. A full list of ICD-10CM codes used is shown in Appendix Table 1. At the time of the search, all 113 HCOs in the Research Network had data available for all cause GN and subtypes and laboratory data for Troponin I and NTproBNP.

**Table 1:** Demographics and CV risk factor profile of all GN cohorts post propensity score matching

	Troponin I				NTproBNP		
**>All Cause GN**							
	**< 18ng/L**	**≥ 18 ng/L**	***P*** **-Value**		**< 400 pg/mL**	**≥ 400 pg/mL**	***P*** **-Value**
Sample Size	17,487	17,487			9,109	9,109	
Age at Index Mean ± SD	59.3 ± 16.9	59.4 ± 17.1	0.592		60.4 ± 16.6	60.1 ± 17.8	0.253
Male N (%)	8,381 (47.9)	8,416 (48.1)	0.708		4,523 (49.7)	4,513 (49.5)	0.882
**Cardiovascular co-morbidities N (%)**							
Hypertension	14,320 (81.9)	14,402 (82.4)	0.252		6,703 (73.6)	6,709 (73.7)	0.920
Ischaemic heart disease	5,463 (31.2)	5,494 (31.4)	0.721		2,879 (31.6)	2,877 (31.6)	0.975
Heart failure	4,153 (23.7)	4,115 (23.5)	0.632		2,008 (22.0)	2,025 (22.2)	0.762
Diabetes mellitus	9,837 (56.3)	9,897 (56.6)	0.518		4,976 (54.6)	4,969 (54.6)	0.917
Smoking	2,931 (16.8)	2,917 (16.7)	0.841		1,264 (13.9)	1,331 (14.6)	0.156
**Cardiovascular medication**							
Beta blockers	10,231 (58.5)	10,313 (59.0)	0.373		4,614 (50.7)	4,762 (52.3)	0.028
Antilipemic agents	9,638 (55.1)	9,690 (55.4)	0.576		4,892 (53.7)	5,011 (55.0)	0.077
Ace inhibitors	7,548 (43.2)	7,564 (43.3)	0.863		3,644 (40.0)	3,684 (40.4)	0.546
Angiotensin II inhibitor	5,184 (29.6)	5,108 (29.2)	0.373		2,705 (29.7)	2,759 (30.3)	0.383
Aspirin	7,439 (42.5)	7,483 (42.8)	0.634		3,997 (43.9)	4,097 (45.0)	0.136
Clopidogrel	1,954 (11.2)	1,983 (11.3)	0.624		1,044 (11.5)	1,088 (11.9)	0.311
Diuretics	10,260 (58.7)	10,286 (58.8)	0.778		4,994 (54.8)	5,118 (56.2)	0.065
Finerenone	10 (0.1)	10 (0.1)	1		10 (0.1)	10 (0.1)	1
Eplerenone	76 (0.4)	76 (0.4)	1		52 (0.6)	50 (0.5)	0.843
Spironolactone	1,663 (9.5)	1,650 (9.4)	0.812		858 (9.4)	838 (9.2)	0.610
**Laboratory results**							
Proteinuria (Microalbumin)							
0–30 mg/g	1,785 (10.2)	1,803 (10.3)	0.751		1,110 (12.2)	1,137 (12.5)	0.543
30–300 mg/g	2,127 (12.2)	2,141 (12.2)	0.819		1,231 (13.5)	1,266 (13.9)	0.451
> 300 mg/g	1,727 (9.9)	1,762 (10.1)	0.532		823 (9.0)	825 (9.1)	0.959
Cholesterol mg/dL	171.8 ± 56.8	174.0 ± 63.3	0.005		177.0 ± 56.7	175.1 ± 62.7	0.072
**IgA Nephropathy**							
	**< 18ng/L**	**≥ 18 ng/L**	**P-Value**		**< 400 pg/mL**	**≥ 400pg/mL**	**P-Value**
Sample Size	6,389	6,389			2,812	2,812	
Age at Index Mean ± SD	55.9 ± 16.6	56.0 ± 17.4	0.745		56.1 ± 17.0	56.0 ± 18.2	0.760
Male N (%)	3,186 (49.9)	3,212 (50.3)	0.646		1,396 (49.6)	1,387 (49.3)	0.810
**Cardiovascular co-morbidities N (%)**							
Hypertension	5,241 (82.0)	5,249 (82.2)	0.854		2,283 (81.2)	2,294 (81.6)	0.706
Ischaemic heart disease	1,759 (27.5)	1,749 (27.4)	0.843		817 (29.1)	815 (29.0)	0.953
Heart failure	1,339 (21.0)	1,310 (20.5)	0.527		595 (21.2)	599 (21.3)	0.896
Diabetes mellitus N (%)	2,827 (44.2)	2,847 (44.6)	0.722		1,278 (45.4)	1,280 (45.5)	0.957
Smoking N (%)	1,139 (17.8)	1,147 (18.0)	0.854		465 (16.5)	477 (17.0)	0.668
**Cardiovascular medication N (%)**							
Beta blockers	3,790 (59.3)	3,796 (59.4)	0.914		1,593 (56.7)	1,642 (58.4)	0.186
Antilipemic agents	3,251 (50.9)	3,270 (51.2)	0.737		1,570 (55.8)	1,615 (57.4)	0.226
Ace inhibitors	2,693 (42.2)	2,692 (42.1)	0.986		1,226 (43.6)	1,235 (43.9)	0.809
Angiotensin II inhibitor	1,887 (29.5)	1,843 (28.8)	0.392		926 (32.9)	958 (34.1)	0.366
Aspirin	2,631 (41.2)	2,655 (41.6)	0.666		1,272 (45.2)	1,310 (46.6)	0.309
Clopidogrel	584 (9.1)	601 (9.4)	0.604		282 (10.0)	290 (10.3)	0.724
Diuretics	3,768 (59.0)	3,757 (58.8)	0.843		1,702 (60.5)	1,752 (62.3)	0.171
Finerenone	10 (0.2)	10 (0.2)	1		10 (0.4)	0 (0)	0.002
Eplerenone	28 (0.4)	31 (0.5)	0.695		17 (0.6)	25 (0.9)	0.215
Spironolactone	574 (9.0)	579 (9.1)	0.877		274 (9.7)	306 (10.9)	0.161
**Laboratory results**							
Proteinuria (Microalbumin)							
0–30 mg/g	383 (6.0)	398 (6.2)	0.580		250 (8.9)	254 (9.0)	0.852
30–300 mg/g	511 (8.0)	527 (8.2)	0.604		300 (10.7)	320 (11.4)	0.394
> 300 mg/g	534 (8.4)	522 (8.2)	0.700		241 (8.6)	262 (9.3)	0.326
Cholesterol mg/dL	174.2 ± 58.1	178.6 ± 67.3	0.002		180.2 ± 60.1	177.4 ± 61.5	0.146
**Membranous Nephropathy**							
	**< 18ng/L**	**≥ 18 ng/L**	**P-Value**		**< 400 pg/mL**	**≥ 400pg/mL**	**P-Value**
Sample Size	5,962	5,962			2,618	2,618	
Age at Index Mean ± SD	56.1 ± 16.7	56.2 ± 17.5	0.795		56.1 ± 17.2	55.5 ± 18.7	0.247
Male N (%)	2,936 (49.2)	2,956 (49.6)	0.714		1,276 (48.7)	1,290 (49.3)	0.699
**Cardiovascular co-morbidities N (%)**							
Hypertension	4,923 (82.6)	4,929 (82.7)	0.885		2,135 (81.6)	2,131 (81.4)	0.887
Ischaemic heart disease	1,673 (28.1)	1,660 (27.8)	0.791		766 (29.3)	759 (29.0)	0.831
Heart failure	1,274 (21.4)	1,236 (20.7)	0.393		563 (21.5)	554 (21.2)	0.761
Diabetes mellitus	2,681 (45.0)	2,684 (45.0)	0.956		1,188 (45.4)	1,189 (45.4)	0.978
Smoking	1,079 (18.1)	1,053 (17.7)	0.534		443 (16.9)	439 (16.8)	0.883
**Cardiovascular medication N (%)**							
Beta blockers	3,566 (59.8)	3,590 (60.2)	0.654		1,488 (56.8)	1,528 (58.4)	0.263
Antilipemic agents	3,097 (51.9)	3,056 (51.3)	0.452		1,470 (56.1)	1,482 (56.6)	0.738
Ace inhibitors	2,578 (43.2)	2,594 (43.5)	0.767		1,168 (44.6)	1,153 (44.0)	0.676
Angiotensin II inhibitor	1,759 (29.5)	1,695 (28.4)	0.196		841 (32.1)	832 (31.8)	0.790
Aspirin	2,495 (41.8)	2,523 (42.3)	0.603		1,215 (46.4)	1,203 (46.0)	0.739
Clopidogrel	556 (9.3)	571 (9.6)	0.639		263 (10.0)	257 (9.8)	0.782
Diuretics	3,565 (59.8)	3,558 (59.7)	0.896		1,613 (61.6)	1,605 (61.3)	0.820
Finerenone	10 (0.2)	10 (0.2)	1		10 (0.4)	10 (0.4)	1
Eplerenone	28 (0.5)	32 (0.5)	0.605		16 (0.6)	14 (0.5)	0.714
Spironolactone	48 (9.2)	518 (8.7)	0.336		262 (10.0)	238 (9.1)	0.259
**Laboratory results**							
Proteinuria (Microalbumin)							
0–30 mg/g	389 (6.5)	388 (6.5)	0.970		243 (9.3)	234 (8.9)	0.666
30–300 mg/g	508 (8.5)	523 (8.8)	0.625		289 (11.0)	285 (10.9)	0.860
> 300 mg/g	516 (8.7)	506 (8.5)	0.744		230 (8.8)	224 (8.6)	0.768
Cholesterol mg/dL	174.6 ± 57.6	178.9 ± 66.1	0.003		180.7 ± 61.2	178.1 ± 60.5	0.197
**Focal Segmental Glomerulosclerosis**							
	**< 18ng/L**	**≥ 18 ng/L**	**P-Value**		**< 400 pg/mL**	**≥ 400pg/mL**	**P-Value**
Sample Size	6,376	6,376			2,810	2,810	
Age at Index Mean ± SD	56.6 ± 16.7	56.6 ± 17.2	0.829		56.4 ± 17.1	56.0 ± 18.8	0.454
Male N (%)	3,157 (49.5)	3,147 (49.4)	0.859		1,362 (48.5)	1,399 (49.8)	0.324
**Cardiovascular co-morbidities N (%)**							
Hypertension	5,232 (82.1)	5,244 (82.2)	0.781		2,290 (81.5)	2,303 (82.0)	0.654
Ischaemic heart disease	1,803 (28.3)	1,819 (28.5)	0.753		816 (29.0)	826 (29.4)	0.769
Heart failure	1,357 (21.3)	1,335 (20.9)	0.633		606 (21.6)	639 (22.7)	0.289
Diabetes mellitus	2,848 (44.7)	2,876 (45.1)	0.618		1,247 (44.4)	1,280 (45.6)	0.376
Smoking	1,169 (18.3)	1,189 (18.6)	0.648		483 (17.2)	504 (17.9)	0.462
**Cardiovascular medication**							
Beta blockers	3,824 (60.0)	3,830 (60.1)	0.914		1,595 (56.8)	1,629 (58.0)	0.359
Antilipemic agents	3,282 (51.5)	3,285 (51.5)	0.958		1,562 (55.6)	1,594 (56.7)	0.390
Ace inhibitors	2,730 (42.8)	2,717 (42.6)	0.816		1,240 (44.1)	1,260 (44.8)	0.591
Angiotensin II inhibitor	1,851 29.0)	1,856 (29.1)	0.922		896 (31.9)	882 (31.4)	0.688
Aspirin	2,676 (42.0)	2,675 (42.0)	0.986		1,313 (46.7)	1,342 (47.8)	0.438
Clopidogrel	578 (9.1)	592 (9.3)	0.668		283 (10.1)	286 (10.2)	0.894
Diuretics	3,795 (59.5)	3,798 (59.6)	0.957		1,744 (62.1)	1,760 (62.6)	0.660
Finerenone	10 (0.2)	10 (0.2)	1		10 (0.4)	10 (0.4)	1
Eplerenone	30 (0.5)	31 (0.5)	0.898		17 (0.6)	20 (0.7)	0.621
Spironolactone	574 (9.0)	576 (9.0)	0.951		286 (10.2)	314 (11.2)	0.226
**Laboratory results**							
Proteinuria (Microalbumin)							
0–30 mg/g	436 (6.8)	415 (6.5)	0.456		263 (9.4)	287 (10.2)	0.281
30–300 mg/g	537 (8.4)	537 (8.4)	1		308 (11.0)	326 (11.6)	0.448
> 300 mg/g	535 (8.4)	547 (8.6)	0.703		233 (8.3)	233 (8.3)	1
Cholesterol mg/dL	174.0 ± 56.6	179.3 ± 66.0	< 0.001		179.9 ± 59.0	176.8 ± 59.9	0.102
**Minimal Change Disease**							
	**< 18ng/L**	**≥ 18 ng/L**	**P-Value**		**< 400 pg/mL**	**≥ 400pg/mL**	**P-Value**
Sample Size	6,561	6,561			3,016	3,016	
Age at Index Mean ± SD	56.7 ± 16.8	56.7 ± 17.5	0.947		56.8 ± 17.3	56.2 ± 19.2	0.206
Male N (%)	3,239 (49.4)	3,260 (49.7)	0.714		1,461 (48.4)	1,494 (49.5)	0.395
**Cardiovascular co-morbidities N (%)**							
Hypertension	5,349 (81.5)	5,296 (80.7)	0.237		2,430 (80.6)	2,422 (80.3)	0.795
Ischaemic heart disease	1,839 (28.0)	1,846 (28.1)	0.892		89 1(29.5)	920 (30.5)	0.415
Heart failure	1,376 (21.0)	1,358 (20.7)	0.699		644 (21.4)	648 (21.5)	0.900
Diabetes mellitus	2,944 (44.9)	2,922 (44.5)	0.699		1,362 (45.2)	1,385 (45.9)	0.552
Smoking	1,189 (18.1)	1,200 (18.3)	0.803		505 (16.7)	500 (16.6)	0.863
**Cardiovascular medication N (%)**							
Beta blockers	3,872 (59.0)	3,845 (58.6)	0.632		1,681 (55.7)	1,695 (56.2)	0.717
Antilipemic agents	3,365 (51.3)	3,339 (50.9)	0.650		1,664 (55.2)	1,655 (54.9)	0.816
Ace inhibitors	2,774 (42.3)	2,772 (42.2)	0.972		1,295 (42.9)	1,311 (43.5)	0.677
Angiotensin II inhibitor	1,867 (28.5)	1,881 (28.7)	0.787		950 (31.5)	937 (31.1)	0.718
Aspirin	2,743 (41.8)	2,708 (41.3)	0.535		1,40 5(46.6)	1,412 (46.8)	0.857
Clopidogrel	601 (9.2)	604 (9.2)	0.928		311 (10.3)	319 (10.6)	0.736
Diuretics	3,888 (59.3)	3,842 (58.6)	0.414		1,831 (60.7)	1,836 (60.9)	0.895
Finerenone	10 (0.2)	10 (0.2)	1		10 (0.3)	10 (0.3)	1
Eplerenone	30 (0.5)	28 (0.4)	0.792		18 (0.6)	22 (0.7)	0.526
Spironolactone	604 (9.2)	597 (9.1)	0.832		306 (10.1)	324 (10.7)	0.449
**Laboratory results**							
Proteinuria (Microalbumin)							
0–30 mg/g	453 (6.9)	443 (6.8)	0.729		283 (9.4)	282 (9.4)	0.965
30–300 mg/g	565 (8.6)	577 (8.8)	0.710		332 (11.0)	332 (11.0)	1
> 300 mg/g	569 (8.7)	590 (9.0)	0.518		256 (8.5)	255 (8.5)	0.963
Cholesterol mg/dL	174.8 ± 58.1	179.2 ± 66.7	0.001		179.6 ± 59.8	176.3 ± 60.6	0.076

Table showing the demographics and CV risk factors for all cause GN and primary GN sub-type cohorts following propensity score matching (PSM). All statistical analysis was performed using the online TriNetX platform. 1:1 PSM using logistic regression. The cohorts were matched for age, gender, comorbidities influencing adverse CV outcomes, cardiac medications and proteinuria at baseline. A *P* < 0.05 was accepted as statistically significant

According to biomarker-specific thresholds, two cohorts were generated for analysis.

1. Troponin I cohorts stratified as Troponin I ≥ 18 ng/L or < 18 ng/L.
2. NT-proBNP cohorts stratified as ≥ 400.00 pg/mL or ˂400.00 pg/mL, respectively.

Cardiac biomarkers were the first reported result within three months of GN diagnosis. The specific thresholds reflect the National Institute of Health and Care Excellence (NICE) guideline for diagnosing heart failure (NTproBNP). Troponin I is an approximation of the 99th percentile across all clinical assay platforms [12].

Demographic data on age and gender were collected, as well as common CV risk factors by ICD-10CM codes, including hypertensive diseases (I10-I16), ischaemic heart disease (IHD) (ICD-10CM: I20-I25), heart failure (ICD-10CM: I50), diabetes mellitus (E08-E13) and smoking status (F17 nicotine dependence). Data was also collected on common cardiovascular medication; beta blockers, antilipemic agents, ace inhibitors, angiotensin II inhibitors, aspirin, clopidogrel, diuretics, finerenone, eplerenone, spironolactone. Laboratory results for estimated glomerular filtration rate (eGFR utilising Modification of Diet in Renal Disease (MDRD) formula)), proteinuria (microalbumin mg/g) and cholesterol (mg/dL) were extracted from the database. Laboratory values were the first reported within three months of GN diagnosis.

### Index Event

The diagnosis of a primary GN with a cardiac biomarker measured within 3 months (NTproBNP or Troponin I) following the diagnosis was used as the index event. The index event whereby a patient meets the criteria for inclusion could be up to 20 years before the data search date.

### Follow-up and clinical outcome

The primary outcome was the incidence of any MACE that occurred between 1 day after the index event and five years follow-up. MACE was defined as a composite of IHD (ICD-10CM: I20-I25), angina (ICD-10CM: I20), acute myocardial infarction (AMI) (MI ICD-10CM: I21), heart failure (ICD-10CM: I50), atrial fibrillation or flutter (ICD-10CM: I48), ischaemic stroke (ICD-10CM: I63), and all-cause mortality (death). Patients who incurred a MACE 5-years prior to the index event were excluded. The secondary outcome was the risk for each component of the composite outcome.

### Statistical analysis

All statistical analyses were performed on the TriNetX online platform. All participants had been enrolled to the database between the years 2010–2024.

As a continuous variable, age was expressed as mean and standard deviation (S.D.) and tested for differences with an independent-sample t-test. The demographic and CV risk factor data were expressed as absolute frequencies and percentages and tested for differences with the chi-squared test.

Prior to analysis, cohorts were 1:1 propensity score matched (PSM) [13] for baseline demographics CV risk factors, CV medications, proteinuria and cholesterol. PSM was performed using the online TriNetX platform. The platform uses ‘greedy nearest-neighbour matching’ with a caliper of 0.1 pooled standard deviations and a difference between propensity scores ≤ 0.1. Covariate balance between groups was assessed using standardised mean differences (SMDs) and included in appendix results, SMD between cohorts < 0.1 is considered well-matched.

Following PSM, Cox proportional hazard models were used to assess the association of cardiac biomarkers with the primary and secondary outcomes at 5-year follow-ups.

Results are reported as hazard ratio HR) with 95% confidence intervals and Kaplan-Meier survival curves with log-rank tests. No imputations were made for missing data. Censoring was applied, and a patient was removed (censored) from the analysis after the last event in their electronic record. Statistical analysis was performed using the’ Analytics’ functionality on TriNetX, which used the R Survival package v3.2-3. A p-value < 0.05 was accepted as the level of statistical significance.

### Exploratory analysis

We performed 3 additional exploratory analyses to understand:

1. The CV risk of patients with GN beyond that attributed to traditional risk factors.
2. The prognostic significance of combining NTproBNP and Troponin I in a single analysis.
3. The prognostic significance of NTproBNP by excluding troponin I and vice-versa.

The first exploratory analysis aimed to study the CV risk of patients with GN beyond that attributed and acknowledged by traditional risk factors such as demographics, comorbidities, CV medication and level of renal function.

We investigated the risk of the primary and secondary outcome in the all-cause GN cohort only following 1:1 PSM, including the same variables as the main analysis with the addition of eGFR.

In the second analysis, we aimed to determine the prognostic utility of a combined biomarker approach, with NTproBNP and Troponin I stratified by their respective thresholds.

In the final analysis, we aimed to determine the prognostic significance of each biomarker (stratified by specific thresholds above) in a population where the alternate biomarker had been reduced.

Both these analyses were performed on the all-cause GN group only following 1:1 PSM including the same variables as the main analysis with the addition of renal function as detailed above. These further 2 exploratory analyses were performed to account for the potential overlap in the populations were NTproBNP and Troponin I are reported.

### Data Access

The data used in this analysis were accessed on the TriNetX online research platform. To gain access to this data a request can be made to TriNetX (https://live.trinetx.com/), although costs may be incurred, and a data sharing agreement must be in place. As a federated research network, studies using TriNetX do not require research ethical approval as no patient’s identifiable information is received.

## Results

### Demographics

#### Troponin I

**Fig. 1:**
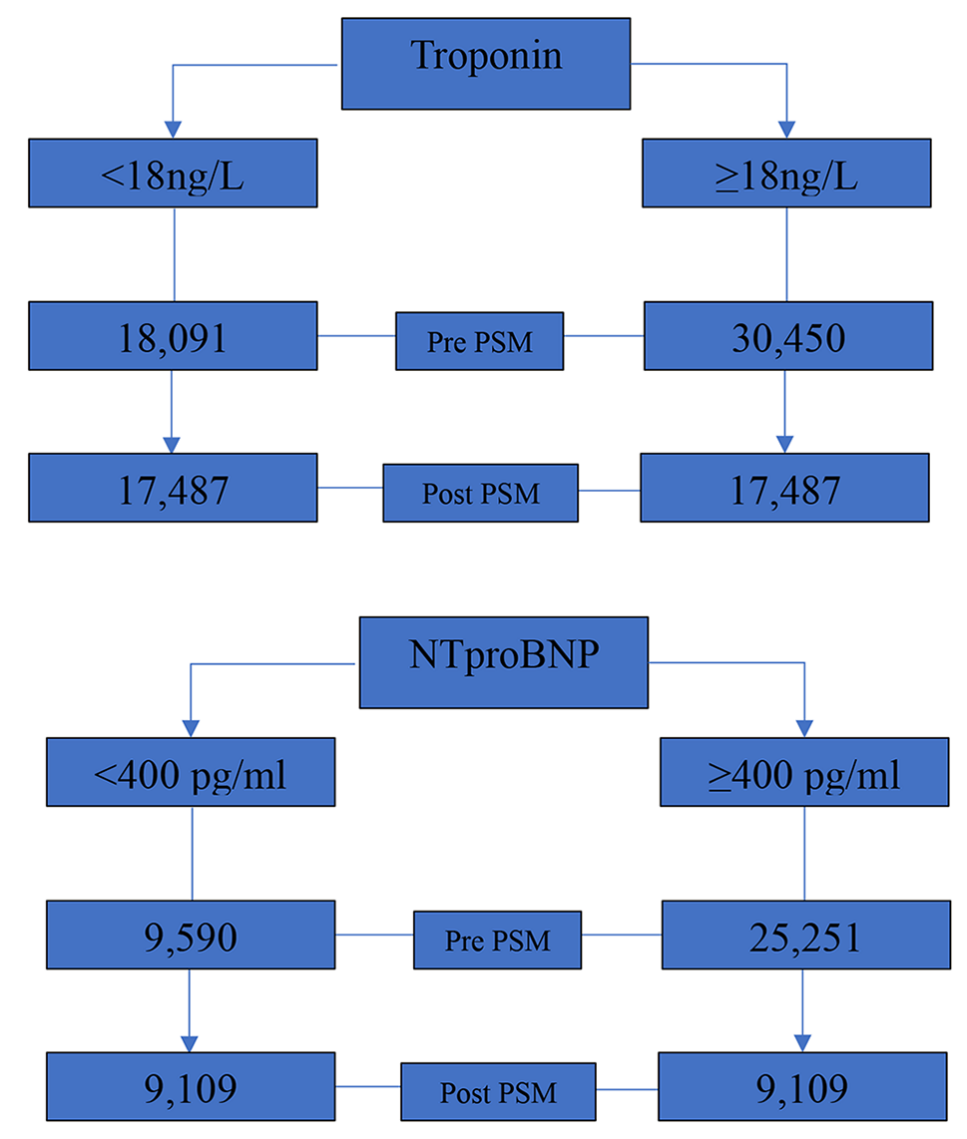
Patient number for pre and post Propensity score matching (PSM) number for Troponin I and NTproBNP all cause GN cohorts. Figure showing the number of patients before and after PSM was applied for all cause GN cohort. Troponin I and NTproBNP cohorts have been separated into their biomarker thresholds for analysis

A total of 48,541 patients with all-cause GN were identified. Prior to propensity score matching (PSM), patients with Troponin I ≥ 18 ng/L were older, a higher proportion of males and a greater prevalence of ischaemic heart disease (IHD), heart failure (HF) and diabetes mellitus. A summary of the PSM characteristics may be found in Appendix Table 2. Following PSM, 34,974 patients were included in the analysis (mean patient age 59.4 SD 17; 48% male). 82% of the cohort patients had hypertension, 31% IHD and 24% HF. Beta-blockers and diuretics were the most common CV medication prescribed at 59%. Across the sub-group analysis, the mean age and CV risk factor profile reflected a similar pattern to all-cause GN. Following PSM, troponin I median and standard deviation (SD) was 75.5 ng/L ± 47.3 vs. 13.6 ng/L ± 1.8, both cohorts (Troponin I < 18 ng/L vs. Troponin I≥18ng/L) were well matched for age, gender and CV risk factors, with no statistically significant differences between groups. A breakdown of patient selection is shown in the study flow diagram. (Fig. 1)

**Table 2:** Demographics and CV risk factor profile post propensity score matching of sub-group adjusted for baseline CKD

	Troponin I				NTproBNP		
**All cause GN**							
	**< 18ng/L**	**≥ 18 ng/L**	***P*** **-Value**		**< 400 pg/mL**	**≥ 400 pg/mL**	***P*** **-Value**
Sample Size	16,911	16,911			8,365	8,365	
Age at Index Mean ± SD	59.5 ± 16.8	59.7 ± 17.1	0.212		60.7 ± 16.8	60.7 ± 18.2	0.858
Male N (%)	8,262 (48.9)	8,221 (48.6)	0.656		4,253 (50.8)	4,311 (51.5)	0.370
**Cardiovascular co-morbidities N (%)**							
Hypertension	13,858 (81.9)	13,798 (81.6)	0.398		6,201 (74.1)	6,168 (73.7)	0.561
Ischaemic heart disease	5,399 (31.9)	5,400 (31.9)	0.991		2,738 (32.7)	2,672 (31.9)	0.275
Heart failure	4,115 (24.3)	4,149 (24.5)	0.667		1,941 (23.2)	1,889 (22.6)	0.339
Diabetes mellitus	9,529 (56.3)	9,570 (56.6)	0.653		4,605 (55.1)	4,586 (54.8)	0.768
Smoking	2,809 (16.6)	2,792 (16.5)	0.804		1,170 (14.0)	1,196 (14.3)	0.564
**Laboratory results**							
eGFR* Mean ± SD	48.5 ± 33.5	42.5 ± 31.9	< 0.001		62.5 ± 31.3	52.6 ± 32.3	< 0.001
**eGFR categories (ml/min/1.73m** ^**2**^ **)**							
> 90	5,169 (30.6)	5,148 (30.4)	0.804		3,532 (42.2)	3,533 (42.2)	0.988
60–89	8,953 (52.9)	8,980 (53.1)	0.769		5,316 (63.6)	5,298 (63.3)	0.773
30–59	10,181 (60.2)	10,258 (60.7)	0.392		4,859 (58.1)	4,994 (59.7)	0.034
15–29	6,840 (40.4)	6,858 (40.6)	0.842		2,236 (26.7)	2,290 (27.4)	0.347
< 15	5,496 (32.5)	5,416 (32.0)	0.352		1,251 (15.0)	1,278 (15.3)	0.560
**Proteinuria (Microalbumin mg/g)**							
0–30	1,698 (10.0)	1,749 (10.3)	0.359		996 (11.9)	1,031 (12.3)	0.407
30–300	2,064 (12.2)	2,134 (12.6)	0.248		1,147 (13.7)	1,204 (14.4)	0.205
> 300	1,675 (9.9)	1,736 (10.3)	0.271		778 (9.3)	797 (9.5)	0.615

Table showing the demographics and CV risk factors for all cause GN following propensity score matching (PSM). All statistical analysis was performed using the online TriNetX platform. 1:1 PSM using logistic regression. The cohorts were matched for age, gender, comorbidities influencing adverse CV outcomes, cardiac medications and proteinuria at baseline and eGFR. A *P* < 0.05 was accepted as statistically significant. *Estimated glomerular filtration rate ml/min/1.73m^2^ (MDRD formula)

#### NT-proBNP

In total, 34,841 patients with all-cause GN were identified. Prior to PSM, patients with NTproBNP ≥ 400 pg/ml were older, a higher proportion male and a greater prevalence of hypertension, IHD and HF. A summary of the PSM characteristics may be found in Appendix Table 3. Following PSM, 18,218 patients were included in the analysis (mean age 60 (SD 17.8); 50% male). Of the all-cause GN cohort, 31.6% had pre-existing HF, 22% IHD and 55% were diabetic. The sub-group analysis of primary GN in this cohort again had similar CV risk factor profiles to all-cause GN. Following PSM NTproBNP median SD was 1204pg/ml ± 803 vs. 183 pg/ml ± 108, both cohorts (NTproBNP < 400 pg/ml vs. NTproBNP ≥400 pg/ml) were well matched for age, gender and CV risk factors, with no statistically significant differences between groups. A breakdown of patient selection is shown in the study flow diagram. (Fig. 1)

Table 1 displays the included patient demographics following PSM and CV risk profile for all GN cohorts.

### Clinical outcomes

#### Troponin I

**Fig. 2:**
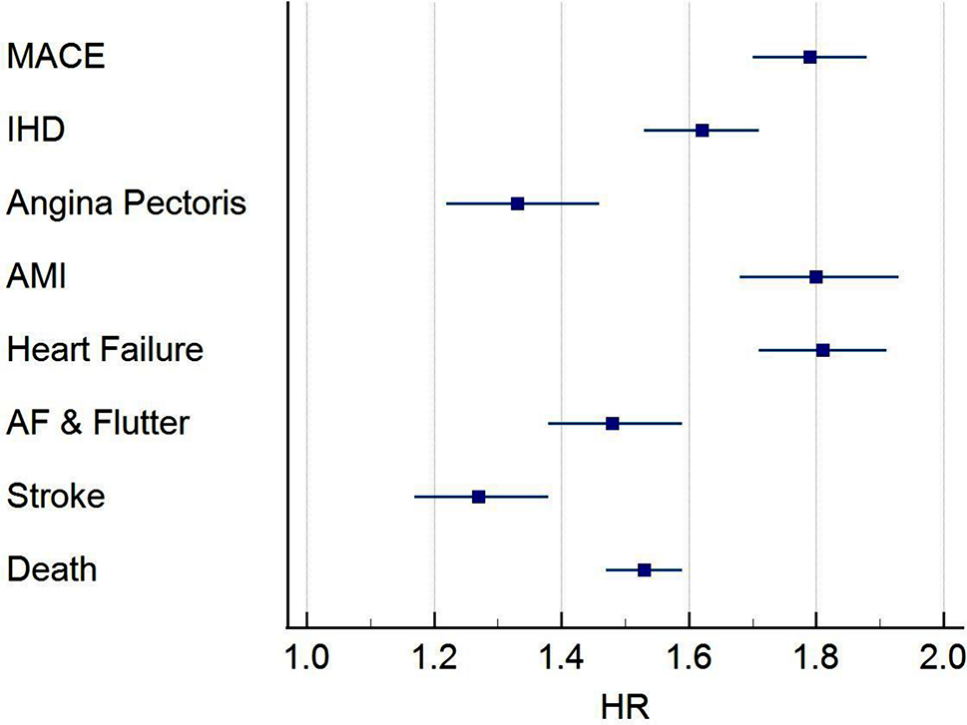
Troponin I and outcome for all cause GN. Forrest Plot shows HR and 95% CI for incident outcome, including composite primary outcome, MACE, and individual components of the primary outcome

Within all-cause GN cohort, 13,625 of the 34,974 patients had 5-year follow-up data available from the time of the index event. Of those, 6,222 developed the primary composite outcome. Of these 3,222 (9% of all-cause GN cohort) had a Troponin I above the 18 ng/L threshold. This equated to a HR of 1.79 (95% CI, 1.70, 1.88, p-value < 0.0001). When considering the secondary outcome, of the individual components of the primary composite outcome, an increased Troponin I was associated with a statistically significant increased risk of all-cause mortality HR 1.53 (95% CI, 1.47, 1.59); stroke HR1.27 (95% CI, 1.17, 1.38); HF HR 1.81 (95% CI, 1.71, 1.91); acute myocardial infarction (AMI) HR 1.79 (95% CI, 1.68, 1.93); angina pectoris HR 1.33 (95% CI, 1.22, 1.46) and IHD HR 1.62 (95% CI, 1.53, 1.71) (Fig. 2). Only atrial fibrillation and flutter as secondary outcomes did not reach the level of statistical significance.

An increased cardiac Troponin I above the 18ng/L threshold was associated with a statistically significant increased risk of the composite primary outcome in all GN sub-groups: IgA nephropathy (IgAN) HR1.75 (95% CI, 1.61, 1.90); membranous nephropathy (MN) HR 1.79 (95% CI, 1.64, 1.94); focal segmental glomerulosclerosis (FSGS) HR 1.71 (95% CI, 1.58, 1.87) and minimal change disease (MCD) HR 1.71 (95% CI, 1.58, 1.86). In the GN sub-groups, the most significant risk associated with an increased cardiac Troponin I was the development of heart failure over the 5 years of follow-up: IgAN HR 1.87 (95% CI, 1.66, 2.10); MN HR 1.90 (95% CI, 1.73, 2.09); FSGS HR 1.84 (95% CI, 1.67, 2.01). Conversely, the risk of AMI correlated most significantly with troponin in MCD HR 1.87 (95% CI, 1.67, 2.01) (Appendix Table 4).

#### NT-proBNP

**Fig. 3:**
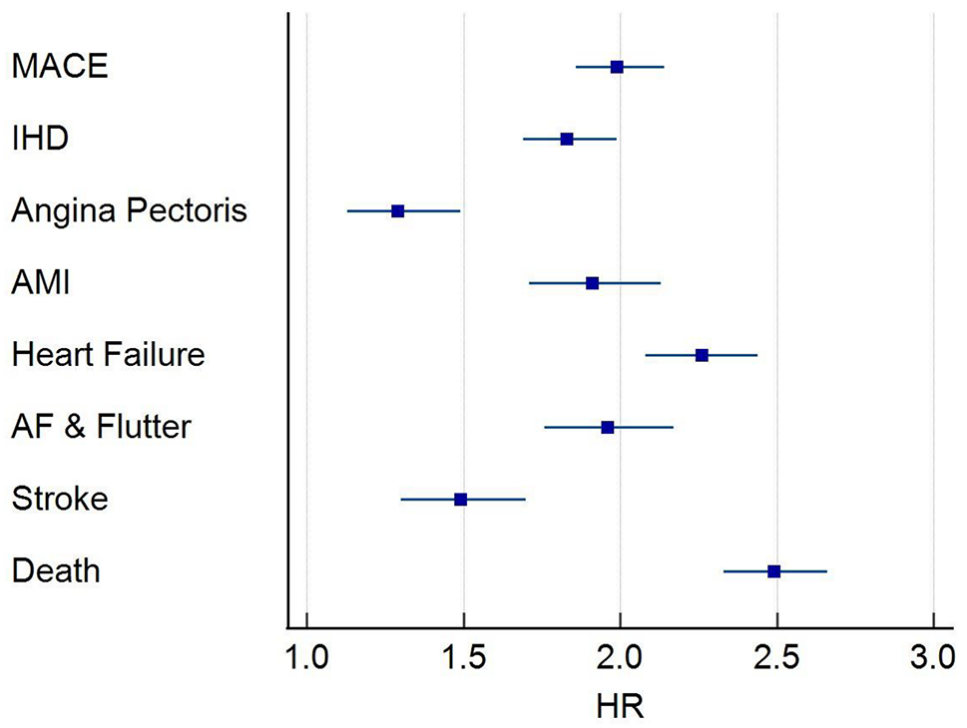
NT-proBNP and outcome for all cause GN. Forrest Plot showing HR and 95% CI for incident outcome including composite primary outcome, MACE and individual components of the primary outcome

Within all-cause GN cohort, 7,116 of the 18,218 patients had 5-year follow-up data available from the time of the index event. Of those, 3,023 developed the primary composite outcome. Of these 1,686 (9% of all-cause GN cohort) had a NTproBNP above the 400 pg/ml threshold. This equated to a HR of 1.99 ((95% CI, 1.86, 2.14, p-value < 0.0001). When considering the secondary outcome, of the individual components of the primary composite outcome, an increased NTproBNP was associated with a statistically significant increased risk of all-cause mortality HR 2.49 (95% CI, 2.33, 2.66); stroke HR 1.49 (95% CI, 1.23, 1.70)); atrial fibrillation and flutter HR 1.96 (95% CI, 1.76, 2.17)); heart failure HR 2.26 (95% CI, 2.08, 2.44); AMI HR 1.91 (95% CI, 1.71, 2.13); and IHD HR 1.83 (95% CI, 1.69, 1.99) (Fig. 3). Only angina pectoris as a secondary outcome did not reach the level of statistical significance.

An increased NTproBNP above the 400 pg/ml threshold was associated with a statistically significant increased risk of the composite primary outcome in all GN sub-groups: IgAN HR 1.84 (95% CI, 1.62, 2.09); MN HR 1.91 (95% CI, 1.68, 2.18); FSGS HR 1.88 (95% CI, 1.65, 2.14) and MCD HR 1.77 (95% CI, 1.56, 2.00). In the GN sub-groups, the most significant risk associated with an increased NTproBNP was HF, over the 5 years of follow-up in: IgAN HR 2.46 (95% CI, 2.11, 2.86) and MN HR 2.43 (95% CI, 2.08, 2.84). A NTproBNP ≥400 pg/ml was most significantly associated with all-cause mortality in FSGS HR 2.406 (95% CI, 2.13, 2.70) and MCD HR 2.41 (95% CI, 2.14, 2.71) (Appendix Table 5).

**Fig. 4:**
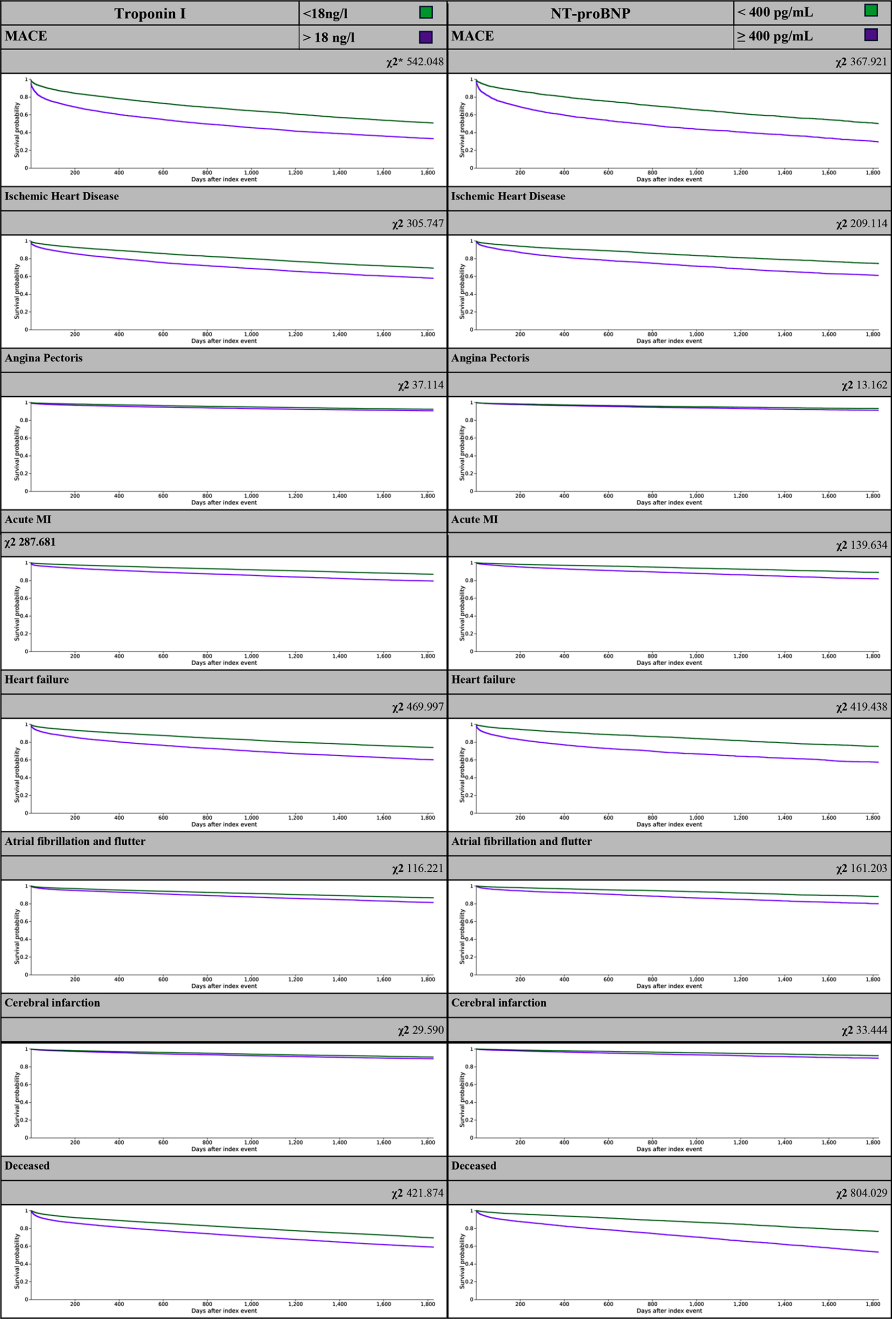
Kaplan - Meier survival analysis for all cause GN cohort. KM for Troponin I and NT-proBNP groups was produced excluding patients with outcome prior to the time window. * χ2 Log-Rank Test

Kaplan - Meier survival analysis (KM) was produced excluding patients with outcome prior to the time window. This analysis highlights that MACE and its components increase the risk of mortality for GN including the sub-group analysis of primary GN (Fig. 4).

### Exploratory analysis- adjusted for baseline CKD

#### Troponin I

**Fig. 5:**
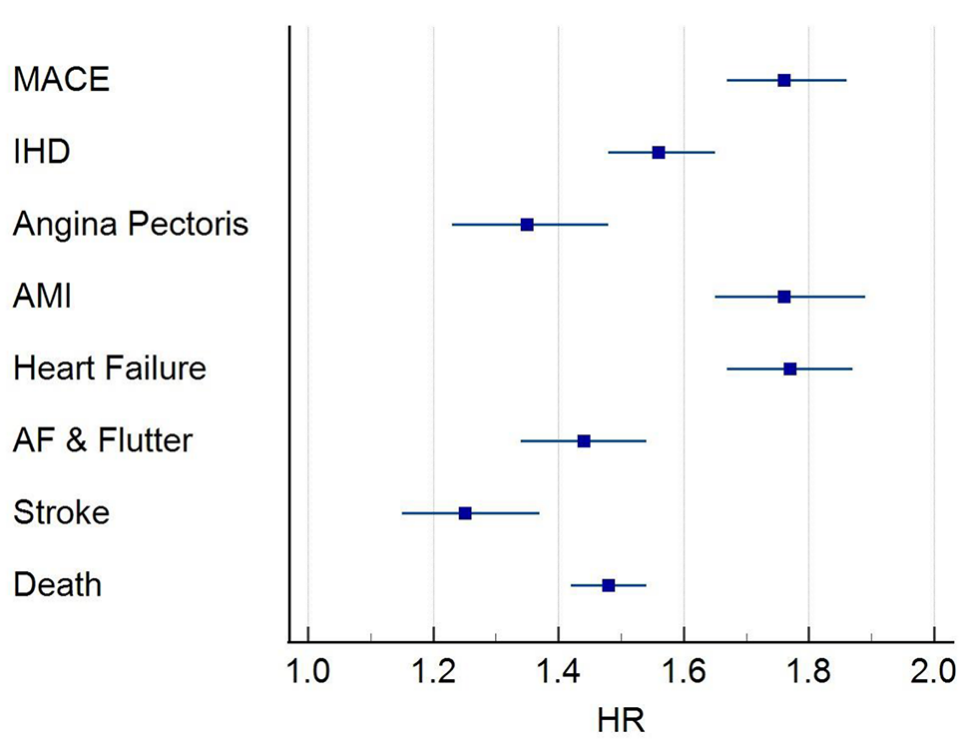
Troponin I and outcome for all cause GN, adjusted for CKD stage. Forrest Plot showing HR and 95% CI for incident outcome including composite primary outcome, MACE and individual components of the primary outcome

In an exploratory analysis, 12,872 of the 33,822 patients had 5-year follow-up data available from the time of the index event. Of those, 5,896 developed the primary composite outcome. Of these, 3,016 (9% of all-cause GN cohort) had a Troponin I above the 18 ng/L threshold. This equated to a HR of 1.76 (95% CI, 1.67,1.86, p-value < 0.0001). When considering the secondary outcome, an increased Troponin I was statistically significant for all components of MACE all-cause mortality HR 1.48 (95% CI, 1.42, 1.54); stroke HR 1.25 (95% CI, 1.15, 1.37); heart failure HR 1.77 (95% CI, 1.67, 1.87); atrial fibrillation and flutter HR 1.44 (1.34, 1.54); AMI HR 1.76 (95% CI, 1.65, 1.89); angina pectoris HR 1.35 (95% CI, 1.23, 1.48) and IHD HR 1.56 (95% CI, 1.48, 1.65) (Fig. 5 and Appendix Table 7).

#### NTproBNP

**Fig. 6:**
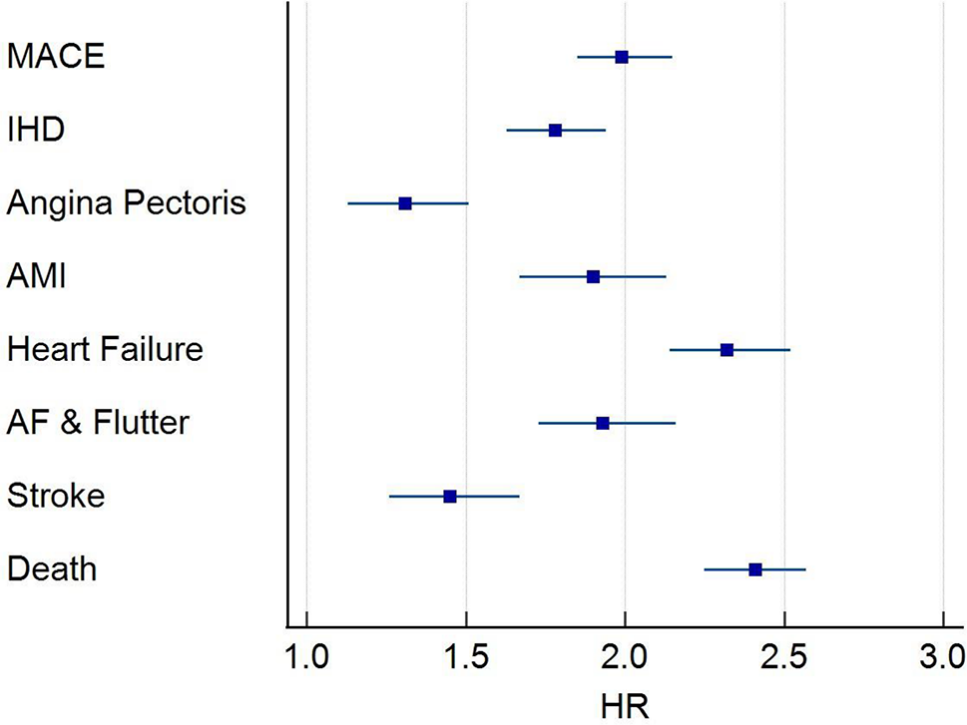
NT-proBNP and outcome for all cause GN, adjusted for CKD stage. Forrest Plot showing HR and 95% CI for incident outcome including composite primary outcome, MACE and individual components of the primary outcome

In our exploratory analysis, 6,333 of the 16,730 patients had 5-year follow-up data available from the time of the index event. Of those, 2,735 developed the primary composite MACE outcome. Of these, 1,500 (9% of all cause GN cohort) had a NTproBNP above the 400 pg/ml threshold. This equated to a HR of 1.99 (95% CI, 1.85, 2.15, p-value < 0.0001). When considering the secondary outcome, an increased NTproBNP was associated with a statistically significant increased risk of all-cause mortality HR 2.41 (95% CI, 2.25, 2.57) ); stroke HR 1.45(95% CI, 1.26, 1.67) ); heart failure HR 2.32 (95% CI, 2.14, 2.52) ; AMI HR 1.90 (95% CI, 1.69, 2.13) ); and IHD HR 1.78 (95% CI, 1.63, 1.94) (Fig. 6). Only angina pectoris as a secondary outcome did not reach the level of statistical significance (Appendix Table 8).

Table 2 displays the included patient demographics following PSM CV risk profile and eGFR for all GN cohorts.

A summary of the PSM characteristics may be found in Appendix Table 6.

### Exploratory analysis – combined NTproBNP and troponin I

In our exploratory analysis of all cause GN with Troponin I and NTproBNP combined, 736 of the 2,318 patients had 5-year follow-up data available from the time of the index event. Of those, 327 developed the primary composite MACE outcome. Of these, 176 (7.6% of all cause GN cohort) had Troponin I and NTproBNP above threshold. This equated to a HR of 2.79 (95% CI, 2.24, 3.48, p-value 0.002). When considering the secondary outcome, statistically significant increased risk was not demonstrated for three of the components of MACE; IHD HR 2.47 (95% CI, 1.96, 3.11,p-value 0.003), AMI HR 3.08 (95% CI, 2.30, 4.12, p-value 0.018), HF HR 2.81 (95% CI, 2.25, 3.51, p-value 0.002). Secondary outcomes that did not meet statistical significance; angina HR 1.69 (95% CI, 1.18, 2.41, p-value 0.893), AF and flutter HR 1.86 (95% CI, 1.38, 2.51, p-value 0.154)stroke HR 1.29 (95% CI, 0.91, 1.81. p-value 0.719), all-cause mortality HR 2.68 (95% CI, 2.25, 3.19, p-value 0.858).

### Exploratory analysis – alternate biomarker excluded

In our exploratory analysis of all cause GN with NTproBNP excluded, 11,339 of the 27,674 patients had 5-year follow-up data available from the time of the index event. Of those, 4,958 developed the primary composite MACE outcome. Of these, 2,608 (9.4% of all cause GN cohort) had a Troponin I above the 18 ng/L threshold. This equated to a HR of 1.81 (95% CI, 1.72, 1.92, p-value < 0.0001). When considering the secondary outcome, statistically significant increased risk was demonstrated for each component of MACE; IHD HR 1.69 (95% CI, 1.59, 1.80,p-value < 0.0001), Angina HR 1.48 (95% CI, 1.32, 1.66, p-value < 0.0001), AMI HR 1.91 (95% CI, 1.76, 2.07, p-value < 0.0001), HF HR 1.94 (95% CI, 1.82, 2.07, p-value < 0.0001), AF and flutter HR 1.61 (95% CI, 1.48, 1.75, p-value 0.003), stroke HR 1.28 (95% CI, 1.16, 1.42. p-value 0.05), all-cause mortality HR 1.51 (95% CI, 1.44, 1.58, p -value < 0.0001).

In our exploratory analysis of all cause GN with Troponin I excluded, 5,250 of the 13,376 patients had 5-year follow-up data available from the time of the index event. Of those, 2,183 developed the primary composite MACE outcome. Of these, 1,244 (9.3% of all cause GN cohort) had a NTproBNP above the 400 pg/ml threshold. This equated to a HR of 1.95(95% CI, 1.79, 2.12, *p* < 0.0001). When considering the secondary outcome, statistically significant increased risk was demonstrated for each component of MACE apart from angina;

IHD HR 1.72 (95% CI, 1.55, 1.90,p-value < 0.0001), AMI HR 1.67 (95% CI, 1.47, 1.91, p-value 0.006), HF HR 2.27 (95% CI, 2.06, 2.50, p-value < 0.0001), AF and flutter HR 2.09 (95% CI, 1.84, 2.38, p-value < 0.0001), stroke HR 1.52 (95% CI, 1.28, 1.79. p-value 0.01), all-cause mortality HR 2.41 (95% CI, 2.23, 2.60, p-value < 0.0001), angina HR 1.36 (95% CI, 1.15, 1.61, p-value 0.7521).

## Discussion

This analysis highlights that routine clinical laboratory cardiac biomarkers, frequently utilised in healthcare settings, can predict incident MACE in patients with GN. Across all GN and sub-groups of primary GN, a raised NT-proBNP and/or Troponin I produced a statistically significant correlation with incident MACE. The exploratory analyses adjusted for baseline CKD demonstrates the CV risk of patients with GN is present beyond the effects conferred by pre-existing traditional risk factors of baseline renal function, in addition to exploring the prognostic significance of a combined biomarker approach.

Multiple studies have recognised the association between circulating plasma cardiac biomarkers and risk of future CV complications in GN patients, however, at present, biomarker monitoring is not a part of standard routine practice for the GN population [14–17]. Our study confirms, in a large study population reflective of real-world clinical use, that Troponin I and NT-proBNP, readily available laboratory tests, provide valuable results that can aid the management of patients with GN.

Proteinuria is synonymous with a GN diagnosis and the correlation between proteinuria and CVD has long been established [18, 19]. For example, Lee et al. [20] conducted a retrospective study of two renal registries analysing patients with biopsy proven membranous nephropathy. One of the measured outcomes was Cardiovascular event (CVE). The study showed a dichotomous pattern of CVE; early events when significant proteinuria and later events over two years since diagnosis not associated with proteinuria. MN disease activity at the time of CVE was a significant independent risk factor HR 2.1, (95% CI, 1.1,4.3) [20]. This highlights that in GN cohorts the pathophysiology leading to CVE can be considered multifactorial; early risk associated with acute immunomodulatory changes and subsequent long-term risk from the GN triggering atherosclerotic pathways.

Ordonez et al. [21] highlighted the increased risk of coronary heart disease associated with nephrotic syndrome (NS) however, we are yet to make significant progress in quantifying and reducing this risk in our GN cohorts. Analysis of data from American electronic health records, The Kaiser Permanente NS Study [22] demonstrated the risk of MACE when comparing a cohort of primary nephrotic patients against a matched adult cohort (adults without diabetes mellitus, NS, or nephrotic range proteinuria). The primary NS cohort demonstrated over a 2.5-fold higher adjusted rate of incident AMI compared with matched controls, adjusted, 2.58 (95% CI, 1.89 to 3.52) [22].

We continue to understand better the pathogenesis of CVD in CKD and the critical role of endothelial dysfunction that may be specific to GN alongside traditional risk factors such as hypertension and dyslipidaemia [23–25]. Biomarkers associated with endothelial dysfunction are present in GN cohorts. Salmito et al. [25] demonstrated a correlation between syndecan-1, a biomarker of endothelial glycocalyx damage, and proteinuria in a cohort of patients with NS. A longitudinal study of patients with FSGS by Zhang et al. [26] showed that the endothelial biomarkers von Willebrand factor and soluble vascular cell adhesion molecule-1 remained elevated despite clinical remission. This study has demonstrated that Troponin I and NTproBNP, validated laboratory tests widely used in clinical practice, can predict the risk of MACE in GN.

NS is associated with dyslipidaemia, including significant hypertriglyceridemia. Persistent dyslipidaemia can exert ‘lipid nephrotoxicity’ [27], which is multifactorial and perpetuates the progression of CKD and subsequent increased risk of CVD [28]. The lipidome of NS patients shows evident dysregulated lipid metabolism, including High-density lipoprotein (HDL) dysfunction. HDL has cardioprotective, antioxidant properties that enhance endothelial function but is dysfunctional in those with CVD disease associated with CKD [3]. Although HDL levels can be measured, no demonstratable threshold can be correlated with increased risk of MACE as we have demonstrated with Troponin I and NTproBNP. There is emerging evidence that the pro-inflammatory process of dyslipidaemia associated with CVD precedes the onset of established CKD [29].

In addition, previous studies in IgAN, the commonest primary GN [30], have aimed to appreciate better and highlight the risk of MACE in this cohort. Based on registry data, Jarrick et al. [31] conducted a retrospective longitudinal analysis of IgAN patients in Sweden. Compared to age and gender-matched cohorts IgAN patients had an increased risk of developing IHD with an adjusted HR 1.86 (95% CI,1.63–2.13). Sagi et al. [32] performed echocardiography prospectively on a cohort of IgAN patients and discovered that the left ventricular mass index could be utilised to predict the risk of mortality, major CV events, and end-stage renal disease. Utilising echocardiography to risk stratify patients requires much more infrastructure and cost compared to routine clinical laboratory measures circulating plasma biomarkers, such as Troponin I and NTproBNP.

The mainstay of treatment for GN is to achieve disease remission using immunosuppressing medication. Patients are frequently exposed to similar levels of immune-modulating medication as transplant patients. Results show that these drugs in themselves can contribute to the development of CV complications [33, 34]. Calcineurin inhibitors (CNI) are common kidney transplant immunosuppression but are also prescribed for GN treatment. CNI has been associated with hypertension in transplant recipients through endothelial dysfunction and oxidative stress; new onset diabetes post-transplantation is also associated with CNI [35–37]. Furthermore, glucocorticoids remain an inherent feature in treatment protocols for GN. Due to the relapsing nature of many GN diagnoses the steroid exposure of a patient can be very significant. Glucocorticoids are associated with hyperglycaemia, hypertension and dyslipidaemia, all well-established risk factors for CVD [38–40].

A study by Hutton et al. [41] based on a prospective Canadian cohort of 2544 patients aimed to examine the hypothesis that the risk of CVD over 3 years in CKD patients with GN is higher than in those with non-GN causes of CKD. The results showed that patients with GN-CKD have a high 8.7% absolute 3-year risk of CVD. However, when the PSM with prior CV risk factors and level of kidney function, the Hazard ratio was 1.01^41^.The first exploratory analysis, reported here, for MACE events adjusted for baseline CKD disproves this theory.

Given the prevalence of GN and CKD and its direct correlation with MACE outcomes, we must identify those individuals at most risk of MACE to address their modifiable risk factors. By virtue of a diagnosis of GN, patients will require frequent monitoring of blood tests. A method can be developed by testing readily available cardiac biomarkers to calculate CV mortality and risk profiling in patients with glomerular disease using a combination of clinical and laboratory variables.

### Strengths and limitations

This study reports a large retrospective cohort of the prognostic significance of routinely measured cardiac biomarkers. The study is based on a large multi-million patient database from participating healthcare organisations. As such the study is reflective of clinical practice. The biomarkers evaluated are already used in clinical practice and can be measured easily in hospital diagnostic laboratories.

While real-world data reflects clinical practice, the retrospective study means the cohorts are not randomised or controlled. However, using a quasi-experimental approach with PSM replicates a randomised control trial within observational data, somewhat mitigating the risk [42]. External validity of the results is limited to the database being studied, this study primarily includes primarily includes participants from North America and Western Europe. The data are derived from electronic health records for administrative purposes; therefore, there is the potential for data errors or missing data. Patients/data may also be lost to follow-up if a patient moves healthcare organisation which could potentially skew covariate distribution and outcomes.

PSM balanced cohorts for age, gender, and CV risk factors. However, omitting socio-economic data such as deprivation indices and family history could bias the results.

## Conclusion

Routinely available cardiac biomarkers can predict incident MACE in patients with GN. The results suggest the clinical need for CV mortality and morbidity risk profiling in patients with glomerular disease using a combination of clinical and laboratory variables.

### Electronic supplementary material

Below is the link to the electronic supplementary material.

Supplementary Material 1

## Data Availability

All data supporting the results reported in the article can be found within the manuscript and the appendix.
