# Supplementary material for: Routine cardiac biomarkers for the prediction of incident major adverse cardiac events in patients with glomerulonephritis: a real-world analysis using a global federated database
Source: BMC Nephrol. 2024 Jul 22;25:233. doi: 10.1186/s12882-024-03667-y (PMC11265111; doi:10.1186/s12882-024-03667-y)
Supplement: Supplementary file 1 — Supplementary Material 1 [file 12882_2024_3667_MOESM1_ESM.docx]

**Appendix**

**Appendix Table 1**: ICD-10CM codes used

| **ICD-10 Clinical Modification Code** | **Description** |
| --- | --- |
| **Glomerulonephritis Cohort** | |
| N00-N08 | Glomerular diseases |
| N05 | Unspecified nephritic syndrome |
| N02.8 | Recurrent and persistent hematuria with other morphologic changes |
| N05.8 | Unspecified nephritic syndrome with other morphologic changes |
| N02.2 | Recurrent and persistent hematuria with diffuse membranous glomerulonephritis |
| N05.2 | Unspecified nephritic syndrome with diffuse membranous glomerulonephritis |
| N04.2 | Nephrotic syndrome with diffuse membranous glomerulonephritis |
| N03.2 | Chronic nephritic syndrome with diffuse membranous glomerulonephritis |
| N00.2 | Acute nephritic syndrome with diffuse membranous glomerulonephritis |
| N05 | Unspecified nephritic syndrome |
| N01.2 | Rapidly progressive nephritic syndrome with diffuse membranous glomerulonephritis |
| N05 | Unspecified nephritic syndrome |
| N05.1 | Unspecified nephritic syndrome with focal and segmental glomerular lesions |
| N04.1 | Nephrotic syndrome with focal and segmental glomerular lesions |
| N06.1 | Isolated proteinuria with focal and segmental glomerular lesions |
| N00.8 | Acute nephritic syndrome with other morphologic changes |
| N03.8 | Chronic nephritic syndrome with other morphologic changes |
| N00 | Acute nephritic syndrome |
| N04.0 | Nephrotic syndrome with minor glomerular abnormality |
| N05.0 | Unspecified nephritic syndrome with minor glomerular abnormality |
| N05 | Unspecified nephritic syndrome |
| N05.8 | Unspecified nephritic syndrome with other morphologic changes |
| N00 | Acute nephritic syndrome |
| N03.8 | Chronic nephritic syndrome with other morphologic changes |
| N00.0 | Acute nephritic syndrome with minor glomerular abnormality |
| N03.0 | Chronic nephritic syndrome with minor glomerular abnormality |
| N02.0 | Recurrent and persistent hematuria with minor glomerular abnormality |
| N01.0 | Rapidly progressive nephritic syndrome with minor glomerular abnormality |
| **IgA Nephropathy** | |
| N05 | Unspecified nephritic syndrome |
| N02.1 | Recurrent and persistent hematuria with focal and segmental glomerular lesions |
| N02.3 | Recurrent and persistent hematuria with diffuse mesangial proliferative glomerulonephritis |
| N02.8 | Recurrent and persistent hematuria with other morphologic changes |
| N02.5 | Recurrent and persistent hematuria with diffuse mesangiocapillary glomerulonephritis |
| N02.2 | Recurrent and persistent hematuria with diffuse membranous glomerulonephritis |
| **Minimal Change Disease** | |
| N05 | Unspecified nephritic syndrome |
| N00 | Acute nephritic syndrome |
| N00.0 | Acute nephritic syndrome with minor glomerular abnormality |
| N03.0 | Chronic nephritic syndrome with minor glomerular abnormality |
| N04.0 | Nephrotic syndrome with minor glomerular abnormality |
| N06.0 | Isolated proteinuria with minor glomerular abnormality |
| N05.0 | Unspecified nephritic syndrome with minor glomerular abnormality |
| N02.0 | Recurrent and persistent hematuria with minor glomerular abnormality |
| N07.0 | Hereditary nephropathy, not elsewhere classified with minor glomerular abnormality |
| N01.0 | Rapidly progressive nephritic syndrome with minor glomerular abnormality |
| **Membranous Glomerulonephritis** | |
| N05 | Unspecified nephritic syndrome |
| N01.2 | Rapidly progressive nephritic syndrome with diffuse membranous glomerulonephritis |
| N02.2 | Recurrent and persistent hematuria with diffuse membranous glomerulonephritis |
| N07.2 | Hereditary nephropathy, not elsewhere classified with diffuse membranous glomerulonephritis |
| **Focal Segmental Glomerulosclerosis** | |
| N05 | Unspecified nephritic syndrome |
| N05.1 | Unspecified nephritic syndrome with focal and segmental glomerular lesions |
| N04.1 | Nephrotic syndrome with focal and segmental glomerular lesions |
| N00.1 | Acute nephritic syndrome with focal and segmental glomerular lesions |
| N02.1 | Recurrent and persistent hematuria with focal and segmental glomerular lesions |
| N01.1 | Rapidly progressive nephritic syndrome with focal and segmental glomerular lesions |
| N03.1 | Chronic nephritic syndrome with focal and segmental glomerular lesions |
| N06.1 | Isolated proteinuria with focal and segmental glomerular lesions |
| N00.8 | Acute nephritic syndrome with other morphologic changes |
| N03.8 | Chronic nephritic syndrome with other morphologic changes |

**Appendix Table 2**. Demographics of all GN Troponin I cohorts and cardiovascular risk factor profile pre and post propensity score matching.

| **TROPONIN I** | | | | | | | |  |
| --- | --- | --- | --- | --- | --- | --- | --- | --- |
|  | **Initial Population (Pre-PSM)** | | | **Propensity Score Matched (PSM) Population** | | | |  |
|  | **<18ng/L** | **≥ 18 ng/L** | **P-value** | | **<18ng/L** | **≥ 18 ng/L** | **P-value** | **SMD** |
| **All Cause GN** | | | | | | | |  |
| **Sample Size (n=)** | 18,091 | 30,450 |  | | 17,487 | 17,487 |  | |
| **Age at Index**  **Mean ± SD** | 58.8  ±17.1 | 61.9  ±16.3 | <0.001 | | 59.3  ± 16.9 | 59.4  ± 17.1 | 0.592 | 0.006 |
| **Male**  **n= (%)** | 8,453  (46.7) | 16,741  (56.2) | <0.001 | | 8,381  (47.9) | 8,416  (48.1) | 0.708 | 0.004 |
| **Cardiovascular co-morbidities n= (%)** | | | | | | | |  |
| **Hypertension** | 14,824  (81.9) | 24,472  (82.2) | 0.449 | | 14,320  (81.9) | 14,402  (82.4) | 0.252 | 0.012 |
| **Ischaemic heart disease** | 5,507  (30.4) | 11,583  (38.9) | <0.001 | | 5,463  (31.2) | 5,494  (31.4) | 0.721 | 0.004 |
| **Heart failure** | 4,166  (23.0) | 9,369  (31.5) | <0.001 | | 4,153  (23.7) | 4,115  (23.5) | 0.632 | 0.005 |
| **Diabetes mellitus** | 10,122  (56.0) | 17,622  (59.2) | <0.001 | | 9,837  (56.3) | 9,897  (56.6) | 0.518 | 0.007 |
| **Smoking** | 3,066  (16.9) | 4,485  (15.1) | <0.001 | | 2,931  (16.8) | 2,917  (16.7) | 0.841 | 0.002 |
| **Cardiovascular medication n= (%)** | | | | | | | |  |
| **Beta blockers** | 10,515  (58.1) | 17,867  (60.0) | <0.001 | | 10,231  (58.5) | 10,313  (59.0) | 0.373 | 0.010 |
| **Antilipemic agents** | 10,034  (55.5) | 15,854  (53.3) | <0.001 | | 9,638  (55.1) | 9,690  (55.4) | 0.576 | 0.006 |
| **Ace inhibitors** | 7,883  (43.6) | 12,164  (40.9) | <0.001 | | 7,548  (43.2) | 7,564  (43.3) | 0.863 | 0.002 |
| **Angiotensin II inhibitor** | 5,429  (30.0) | 7,984  (26.8) | <0.001 | | 5,184  (29.6) | 5,108  (29.2) | 0.373 | 0.010 |
| **Aspirin** | 7,718  (42.7) | 12,623  (42.4) | 0.585 | | 7,439  (42.5) | 7,483  (42.8) | 0.634 | 0.005 |
| **Clopidogrel** | 1,978  (10.9) | 3,849  (12.9) | <0.001 | | 1,954  (11.2) | 1,983  (11.3) | 0.624 | 0.005 |
| **Diuretics** | 10,583  (58.5) | 17,744  (59.6) | 0.016 | | 10,260  (58.7) | 10,286  (58.8) | 0.778 | 0.003 |
| **Finerenone** | 10  (0.1) | 10  (0.0) | 0.260 | | 10  (0.1) | 10  (0.1) | 1 | <0.001 |
| **Eplerenone** | 77  (0.4) | 139  (0.5) | 0.513 | | 76  (0.4) | 76  (0.4) | 1 | <0.001 |
| **Spironolactone** | 1,720  (9.5) | 2,799  (9.4) | 0.706 | | 1,663  (9.5) | 1,650  (9.4) | 0.812 | 0.003 |
| **Laboratory results** | | | | | | | |  |
| **eGFR categories (ml/min/1.73m^2^)** | | | | | | | | |
| **>90** | 6,020  (33.3) | 6,597  (22.2) | <0.001 | | 5,708  (32.6) | 4,267  (24.4) | <0.001 | 0.183 |
| **60-89** | 9,987  (55.2) | 12,432  (41.8) | <0.001 | | 9,586  (54.8) | 7,731  (44.2) | <0.001 | 0.213 |
| **30-59** | 10,887  (60.2) | 17,083  (57.4) | <0.001 | | 10,518  (60.1) | 10,274  (58.8) | 0.008 | 0.028 |
| **15-29** | 7,031  (38.9) | 13,911  (46.7) | <0.001 | | 6,793  (38.8) | 8,222  (47.0) | <0.001 | 0.166 |
| **< 15** | 5,636  (31.2) | 11,623  (39.0) | <0.001 | | 5,436  (31.1) | 6,985  (39.9) | <0.001 | 0.186 |
| **Proteinuria (Microalbumin mg/g)** | | | | | | | | |
| **0 - 30** | 1,953  (10.8) | 2,319  (7.8) | <0.001 | | 1,785  (10.2) | 1,803  (10.3) | 0.751 | 0.003 |
| **30 - 300** | 2,241  (12.4) | 3,280  (11.0) | <0.001 | | 2,127  (12.2) | 2,141  (12.2) | 0.819 | 0.002 |
| **>300** | 1,766  (9.8) | 3,049  (10.2) | 0.090 | | 1,727  (9.9) | 1,762  (10.1) | 0.532 | 0.007 |
| **Cholesterol mg/dL (Mean ± SD)** | 172.2  ± 57.1 | 170.4 ±  61.5 | 0.008 | | 171.8  ± 56.8 | 174.0  ± 63.3 | 0.005 | 0.037 |
|  | | | | | | | |  |
| **IgA Nephropathy** | | | | | | | |  |
| **Sample Size (n=)** | 6,867 | 10,610 |  | | 6,389 | 6,389 |  | |
| **Age at Index**  **Mean ± SD** | 55.2  ± 16.9 | 59.2  ± 16.8 | <0.001 | | 55.9  ± 16.6 | 56.0  ± 17.4 | 0.745 | 0.006 |
| **Male**  **n= (%)** | 3,271  (47.6) | 6,032  (57.4) | <0.001 | | 3,186  (49.9) | 3,212  (50.3) | 0.646 | 0.008 |
| **Cardiovascular co-morbidities n= (%)** | | | | | | | |  |
| **Hypertension** | 5,649  (82.3) | 8,706  (82.9) | 0.274 | | 5,241  (82.0) | 5,249  (82.2) | 0.854 | 0.003 |
| **Ischaemic heart disease** | 1,790  (26.1) | 3,813  (36.3) | <0.001 | | 1,759  (27.5) | 1,749  (27.4) | 0.843 | 0.004 |
| **Heart failure** | 1,347  (19.6) | 3,110  (29.6) | <0.001 | | 1,339  (21.0) | 1,310  (20.5) | 0.527 | 0.011 |
| **Diabetes mellitus** | 3,019  (44.0) | 5,086  (48.4) | <0.001 | | 2,827  (44.2) | 2,847  (44.6) | 0.722 | 0.006 |
| **Smoking** | 1,242  (18.1) | 1,763  (16.8) | 0.027 | | 1,139  (17.8) | 1,147  (18.0) | 0.854 | 0.003 |
| **Cardiovascular medication n= (%)** | | | | | | | |  |
| **Beta blockers** | 4,045  (58.9) | 6,397  (60.9) | 0.008 | | 3,790  (59.3) | 3,796  (59.4) | 0.914 | 0.002 |
| **Antilipemic agents** | 3,565  (51.9) | 5,244  (49.9) | 0.011 | | 3,251  (50.9) | 3,270  (51.2) | 0.737 | 0.006 |
| **Ace inhibitors** | 2,984  (43.5) | 4,124  (39.3) | <0.001 | | 2,693  (42.2) | 2,692  (42.1) | 0.986 | <0.001 |
| **Angiotensin II inhibitor** | 2,090  (30.4) | 2,819  (26.8) | <0.001 | | 1,887  (29.5) | 1,843  (28.8) | 0.392 | 0.015 |
| **Aspirin** | 2,884  (42.0) | 4,267  (40.6) | 0.074 | | 2,631  (41.2) | 2,655  (41.6) | 0.666 | 0.008 |
| **Clopidogrel** | 605  (8.8) | 1,184  (11.3) | <0.001 | | 584  (9.1) | 601  (9.4) | 0.604 | 0.009 |
| **Diuretics** | 4,052  (59.0) | 6,224  (59.3) | 0.730 | | 3,768  (59.0) | 3,757  (58.8) | 0.843 | 0.003 |
| **Finerenone** | 10  (0.1) | 10  (0.1) | 0.338 | | 10  (0.2) | 10  (0.2) | 1 | <0.001 |
| **Eplerenone** | 31  (0.5) | 54  (0.5) | 0.562 | | 28  (0.4) | 31  (0.5) | 0.695 | 0.007 |
| **Spironolactone** | 617  (9.0) | 927  (8.8) | 0.722 | | 574  (9.0) | 579  (9.1) | 0.877 | 0.003 |
| **Laboratory results** | | | | | | | |  |
| **eGFR categories (ml/min/1.73m^2^)** | | | | | | | | |
| **>90** | 2,242  (32.6) | 2,203  (21.0) | <0.001 | | 1,995  (31.2) | 1,566  (24.5) | <0.001 | 0.150 |
| **60-89** | 3,625  (52.8) | 4,214  (40.1) | <0.001 | | 3,299  (51.6) | 2,821  (44.2) | <0.001 | 0.150 |
| **30-59** | 4,069  (59.3) | 5,890  (56.1) | <0.001 | | 3,757  (58.8) | 3,669  (57.4) | 0.115 | 0.028 |
| **15-29** | 2,881  (42.0) | 5,209  (49.6) | <0.001 | | 2,681  (42.0) | 3,158  (49.4) | <0.001 | 0.150 |
| **< 15** | 2,558  (37.3) | 4,734  (45.1) | <0.001 | | 2,383  (37.3) | 2,958  (46.3) | <0.001 | 0.183 |
| **Proteinuria (Microalbumin mg/g)** | | | | | | | | |
| **0 - 30** | 507  (7.4) | 473  (4.5) | <0.001 | | 383  (6.0) | 398  (6.2) | 0.580 | 0.010 |
| **30 - 300** | 605  (8.8) | 774  (7.4) | 0.001 | | 511  (8.0) | 527  (8.2) | 0.604 | 0.009 |
| **>300** | 583  (8.5) | 893  (8.5) | 0.974 | | 534  (8.4) | 522  (8.2) | 0.700 | 0.007 |
| **Cholesterol mg/dL (Mean ± SD)** | 175.3  ± 58.2 | 174.3  ± 64.8 | 0.439 | | 174.2  ± 58.1 | 178.6  ± 67.3 | 0.002 | 0.069 |
|  | | | | | | | |  |
| **Membranous Nephropathy** | | | | | | | |  |
| **Sample Size (n=)** | 6,405 | 10,034 |  | | 5,962 | 5,962 |  | |
| **Age at Index**  **Mean ± SD** | 55.3  ± 17.0 | 59.4  ± 16.8 | <0.001 | | 56.1  ± 16.7 | 56.2  ± 17.5 | 0.795 | 0.005 |
| **Male**  **n= (%)** | 2,996  (46.8) | 5,635  (56.8) | <0.001 | | 2,936  (49.2) | 2,956  (49.6) | 0.714 | 0.007 |
| **Cardiovascular co-morbidities n= (%)** | | | | | | | |  |
| **Hypertension** | 5,292  (82.6) | 8,249  (83.1) | 0.416 | | 4,923  (82.6) | 4,929  (82.7) | 0.885 | 0.003 |
| **Ischaemic heart disease** | 1,695  (26.5) | 3,642  36.7) | <0.001 | | 1,673  (28.1) | 1,660  (27.8) | 0.791 | 0.005 |
| **Heart failure** | 1,279  (20.0) | 2,974  (30.0) | <0.001 | | 1,274  (21.4) | 1,236  (20.7) | 0.393 | 0.016 |
| **Diabetes mellitus** | 2,860  (44.7) | 4,902  (49.4) | <0.001 | | 2,681  (45.0) | 2,684  (45.0) | 0.956 | 0.001 |
| **Smoking** | 1,174  (18.3) | 1,671  (16.8) | 0.014 | | 1,079  (18.1) | 1,053  (17.7) | 0.534 | 0.011 |
| **Cardiovascular medication n= (%)** | | | | | | | |  |
| **Beta blockers** | 3,812  (59.5) | 6,067  (61.1) | 0.040 | | 3,566  (59.8) | 3,590  (60.2) | 0.654 | 0.008 |
| **Antilipemic agents** | 3,366  (52.6) | 4,994  (50.3) | 0.005 | | 3,097  (51.9) | 3,056  (51.3) | 0.452 | 0.014 |
| **Ace inhibitors** | 2,850  (44.5) | 3,961  (39.9) | <0.001 | | 2,578  (43.2) | 2,594  (43.5) | 0.767 | 0.005 |
| **Angiotensin II inhibitor** | 1,945  (30.4) | 2,671  (26.9) | <0.001 | | 1,759  (29.5) | 1,695  (28.4) | 0.196 | 0.024 |
| **Aspirin** | 2,728  (42.6) | 4,092  (41.2) | 0.085 | | 2,495  (41.8) | 2,523  (42.3) | 0.603 | 0.010 |
| **Clopidogrel** | 571  (8.9) | 1,135  (11.4) | <0.001 | | 556  (9.3) | 571  (9.6) | 0.639 | 0.009 |
| **Diuretics** | 3,833  (59.8) | 5,935  (59.8) | 0.954 | | 3,565  (59.8) | 3,558  (59.7) | 0.896 | 0.002 |
| **Finerenone** | 10  (0.2) | 10  (0.1) | 0.323 | | 10  (0.2) | 10  (0.2) | 1 | <0.001 |
| **Eplerenone** | 29  (0.5) | 50  (0.5) | 0.646 | | 28  (0.5) | 32  (0.5) | 0.605 | 0.009 |
| **Spironolactone** | 584  (9.1) | 894  (9.0) | 0.810 | | 48  (9.2) | 518  (8.7) | 0.336 | 0.018 |
| **Laboratory results** | | | | | | | |  |
| **eGFR categories (ml/min/1.73m^2^)** | | | | | | | | |
| **>90** | 2,140  (33.4) | 2,113  (21.3) | <0.001 | | 1,931  (32.4) | 1,442  (24.2) | <0.001 | 0.183 |
| **60-89** | 3,438  (53.7) | 4,025  (40.6) | <0.001 | | 3,152  (52.9) | 2,616  (43.9) | <0.001 | 0.181 |
| **30-59** | 3,833  (59.8) | 5,645  (56.9) | <0.001 | | 3,556  (59.6) | 3,487  (58.5) | 0.199 | 0.024 |
| **15-29** | 2,691  (42.0) | 4,966  (50.0) | <0.001 | | 2,510  (42.1) | 3,008  (50.5) | <0.001 | 0.168 |
| **< 15** | 2,391  (37.3) | 4,462  (45.0) | <0.001 | | 2,223  (37.3) | 2,759  (46.3) | <0.001 | 0.183 |
| **Proteinuria (Microalbumin mg/g)** | | | | | | | |  |
| **0 - 30** | 93  (7.7) | 466  (4.7) | <0.001 | | 389  (6.5) | 388  (6.5) | 0.970 | 0.001 |
| **30 - 300** | 579  (9.0) | 760  (7.7) | 0.002 | | 508  (8.5) | 523  (8.8) | 0.625 | 0.009 |
| **>300** | 553  (8.6) | 865  (8.7) | 0.857 | | 516  (8.7) | 506  (8.5) | 0.744 | 0.006 |
| **Cholesterol mg/dL (Mean ± SD)** | 175.8  ± 58.6 | 174.7  ± 65.2 | 0.369 | | 174.6  ± 57.6 | 178.9  ± 66.1 | 0.003 | 0.069 |
|  | | | | | | | |  |
| **Focal segmental Glomerulosclerosis** | | | | | | | |  |
| **Sample Size (n=)** | 6,821 | 10,667 |  | | 6,376 | 6,376 |  | |
| **Age at Index**  **Mean ± SD** | 55.8 ± 16.9 | 59.6 ± 16.7 | <0.001 | | 56.6  ± 16.7 | 56.6  ± 17.2 | 0.829 | 0.004 |
| **Male**  **n= (%)** | 3,223  (47.3) | 5,976  (56.7) | <0.001 | | 3,157  (49.5) | 3,147  (49.4) | 0.859 | 0.003 |
| **Cardiovascular co-morbidities n= (%)** | | | | | | | |  |
| **Hypertension** | 5,598  (82.1) | 8,746  (82.9) | 0.151 | | 5,232  (82.1) | 5,244  (82.2) | 0.781 | 0.005 |
| **Ischaemic heart disease** | 1,828  (26.8) | 3,884  (36.8) | <0.001 | | 1,803  (28.3) | 1,819  (28.5) | 0.753 | 0.006 |
| **Heart failure** | 1,367  (20.0) | 3,141  (29.8) | <0.001 | | 1,357  (21.3) | 1,335  (20.9) | 0.633 | 0.008 |
| **Diabetes mellitus** | 3,037  (44.5) | 5,165  (49.0) | <0.001 | | 2,848  (44.7) | 2,876  (45.1) | 0.618 | 0.009 |
| **Smoking** | 1,270  (18.6) | 1,798  (17.0) | 0.008 | | 1,169  (18.3) | 1,189  (18.6) | 0.648 | 0.008 |
| **Cardiovascular medication n= (%)** | | | | | | | |  |
| **Beta blockers** | 4,061  (59.5) | 6,440  (61.1) | 0.046 | | 3,824  (60.0) | 3,830  (60.1) | 0.914 | 0.002 |
| **Antilipemic agents** | 3,557  (52.1) | 5,299  (50.2) | 0.014 | | 3,282  (51.5) | 3,285  (51.5) | 0.958 | 0.001 |
| **Ace inhibitors** | 2,989  (43.8) | 4,193  (39.8) | <0.001 | | 2,730  (42.8) | 2,717  (42.6) | 0.816 | 0.004 |
| **Angiotensin II inhibitor** | 2,030  (29.8) | 2,830  (26.8) | <0.001 | | 1,851  29.0) | 1,856  (29.1) | 0.922 | 0.002 |
| **Aspirin** | 2,908  (42.6) | 4,335  (41.1) | 0.045 | | 2,676  (42.0) | 2,675  (42.0) | 0.986 | <0.001 |
| **Clopidogrel** | 597  (8.8) | 1,188  (11.3) | <0.001 | | 578  (9.1) | 592  (9.3) | 0.668 | 0.008 |
| **Diuretics** | 4,068  (59.6) | 6,304  (59.8) | 0.869 | | 3,795  (59.5) | 3,798  (59.6) | 0.957 | 0.001 |
| **Finerenone** | 10  (0.1) | 10  (0.1) | 0.326 | | 10  (0.2) | 10  (0.2) | 1 | <0.001 |
| **Eplerenone** | 32  (0.5) | 53  (0.5) | 0.759 | | 30  (0.5) | 31  (0.5) | 0.898 | 0.002 |
| **Spironolactone** | 618  (9.1) | 941  (8.9) | 0.754 | | 574  (9.0) | 576 (9.0) | 0.951 | 0.001 |
| **Laboratory results** | | | | | | | |  |
| **eGFR categories (ml/min/1.73m^2^)** | | | | | | | | |
| **>90** | 2,310  (33.9) | 2,270  (21.5) | <0.001 | | 2,095  (32.9) | 1,537  (24.1) | <0.001 | 0.195 |
| **60-89** | 3,711  (54.4) | 4,319  (40.9) | <0.001 | | 3,415  (53.6) | 2,827  (44.3) | <0.001 | 0.185 |
| **30-59** | 4,136  (60.6) | 6,062  (57.5) | <0.001 | | 3,848  (60.4) | 3,751  (58.8) | 0.080 | 0.031 |
| **15-29** | 2,902  (42.5) | 5,336  (50.6) | <0.001 | | 2,700  (42.3) | 3,259  (51.1) | <0.001 | 0.176 |
| **< 15** | 2,513  (36.8) | 4,732  (44.9) | <0.001 | | 2,328  (36.5) | 2,942  (46.1) | <0.001 | 0.197 |
| **Proteinuria (Microalbumin mg/g)** | | | | | | | | |
| **0 - 30** | 540  (7.9) | 515  (4.9) | <0.001 | | 436  (6.8) | 415  (6.5) | 0.456 | 0.013 |
| **30 - 300** | 605  (8.9) | 810  (7.7) | 0.005 | | 537  (8.4) | 537  (8.4) | 1 | <0.001 |
| **>300** | 580  (8.5) | 900  (8.5) | 0.946 | | 535  (8.4) | 547  (8.6) | 0.703 | 0.007 |
| **Cholesterol mg/dL (Mean ± SD)** | 175.0  ± 57.1 | 173.6  ± 63.4 | 0.225 | | 174.0  ± 56.6 | 179.3  ± 66.0 | <0.001 | 0.087 |
|  | | | | | | | |  |
| **Minimal Change Disease** | | | | | | | |  |
| **Sample Size (n=)** | 7,075 | 10,878 |  | | 6,561 | 6,561 |  | |
| **Age at Index**  **Mean ± SD** | 55.8  ± 17.1 | 59.7  ± 16.8 | <0.001 | | 56.7  ± 16.8 | 56.7  ± 17.5 | 0.947 | 0.001 |
| **Male**  **n= (%)** | 3,325  (47.0) | 6,058  (56.4) | <0.001 | | 3,239  (49.4) | 3,260  (49.7) | 0.714 | 0.006 |
| **Cardiovascular co-morbidities n= (%)** | | | | | | | |  |
| **Hypertension** | 5,761  (81.4) | 8,844  (82.3) | 0.131 | | 5,349  (81.5) | 5,296  (80.7) | 0.237 | 0.021 |
| **Ischaemic heart disease** | 1,873  (26.5) | 3,926  (36.5) | <0.001 | | 1,839  (28.0) | 1,846  (28.1) | 0.892 | 0.002 |
| **Heart failure** | 1,396  (19.7) | 3,185  (29.6) | <0.001 | | 1,376  (21.0) | 1,358  (20.7) | 0.699 | 0.007 |
| **Diabetes mellitus** | 3,142  (44.4) | 5,275  (49.1) | <0.001 | | 2,944  (44.9) | 2,922  (44.5) | 0.699 | 0.007 |
| **Smoking** | 1,310  (18.5) | 1,793  (16.7) | 0.002 | | 1,189  (18.1) | 1,200  (18.3) | 0.803 | 0.004 |
| **Cardiovascular medication n= (%)** | | | | | | | |  |
| **Beta blockers** | 4,144  (58.6) | 6,517  (60.7) | 0.005 | | 3,872  (59.0) | 3,845  (58.6) | 0.632 | 0.008 |
| **Antilipemic agents** | 3,670  (51.9) | 5,387  (50.1) | 0.024 | | 3,365  (51.3) | 3,339  (50.9) | 0.650 | 0.008 |
| **Ace inhibitors** | 3,069  (43.4) | 4,257  (39.6) | <0.001 | | 2,774  (42.3) | 2,772  (42.2) | 0.972 | 0.001 |
| **Angiotensin II inhibitor** | 2,085  (29.5) | 2,848  (26.5) | <0.001 | | 1,867  (28.5) | 1,881  (28.7) | 0.787 | 0.005 |
| **Aspirin** | 2,988  (42.2) | 4,384  (40.8) | 0.058 | | 2,743  (41.8) | 2,708  (41.3) | 0.535 | 0.011 |
| **Clopidogrel** | 623  (8.8) | 1,209  (11.3) | <0.001 | | 601 (9.2) | 604  (9.2) | 0.928 | 0.002 |
| **Diuretics** | 4,193  (59.3) | 6,395  (59.5) | 0.733 | | 3,888  (59.3) | 3,842  (58.6) | 0.414 | 0.014 |
| **Finerenone** | 10  (0.1) | 10  (0.1) | 0.346 | | 10  (0.2) | 10  (0.2) | 1 | <0.001 |
| **Eplerenone** | 30  (0.4) | 53  (0.5) | 0.506 | | 30  (0.5) | 28  (0.4) | 0.792 | 0.005 |
| **Spironolactone** | 642  (9.1) | 972  (9.0) | 0.950 | | 604  (9.2) | 597  (9.1) | 0.832 | 0.004 |
| **Laboratory results** | | | | | | | |  |
| **eGFR categories (ml/min/1.73m^2^)** | | | | | | | | |
| **>90** | 2,489  (35.2) | 2,369  (22.0) | <0.001 | | 2,221  (33.9) | 1,652  (25.2) | <0.001 | 0.191 |
| **60-89** | 3,913  (55.3) | 4,474  (41.6) | <0.001 | | 3,587  (54.7) | 2,962  (45.1) | <0.001 | 0.191 |
| **30-59** | 4,238  (59.9) | 6,181  (57.5) | 0.002 | | 3,939  (60.0) | 3,871  (59.0) | 0.227 | 0.021 |
| **15-29** | 2,914  (41.2) | 5,322  (49.5) | <0.001 | | 2,716  (41.4) | 3,204  (48.8) | <0.001 | 0.150 |
| **< 15** | 2,511  (35.5) | 4,678  (43.5) | <0.001 | | 2,326  (35.5) | 2,875  (43.8) | <0.001 | 0.172 |
| **Proteinuria (Microalbumin mg/g)** | | | | | | | | |
| **0 - 30** | 568  (8.0) | 526  (4.9) | <0.001 | | 453  (6.9) | 443  (6.8) | 0.729 | 0.006 |
| **30 - 300** | 640  (9.0) | 826  (7.7) | 0.001 | | 565  (8.6) | 577  (8.8) | 0.710 | 0.006 |
| **>300** | 612  (8.7) | 933  (8.7) | 0.938 | | 569  (8.7) | 590  (9.0) | 0.518 | 0.011 |
| **Cholesterol mg/dL (Mean ± SD)** | 175.7  ± 58.8 | 173.9  ± 64.0 | 0.146 | | 174.8  ± 58.1 | 179.2  ± 66.7 | 0.001 | 0.070 |

Table showing the demographics and cardiovascular risk factors for all cause GN and primary GN sub-type cohorts for Troponin I. Results prior to and following propensity score matching (PSM) are included. All statistical analysis was performed using the online TriNetX platform. 1:1PSM using logistic regression. The cohorts were matched for age, sex, and comorbidities influencing adverse CV outcomes (hypertension, heart failure diabetes mellitus and smoking status) at baseline. A P<0.05 was accepted as statistically significant.

**Appendix Table 3**. Demographics of all GN NTproBNP cohorts and cardiovascular risk factor profile pre and post propensity score matching.

| **NTproBNP** | | | | | | | |  |
| --- | --- | --- | --- | --- | --- | --- | --- | --- |
|  | **Initial Population (Pre-PSM)** | | | **Propensity Score Matched (PSM) Population** | | | |  |
|  | **NTproBNP <400 pg/ml** | **NTproBNP**  **≥400 pg/ml** | **P-value** | | **NTproBNP <400 pg/ml** | **NTproBNP**  **≥400 pg/ml** | **P-value** | **SMD** |
| **All Cause GN** | | | | | | | |  |
| **Sample Size (n=)** | 9,590 | 25,251 |  | | 9,109 | 9,109 |  | |
| **Age at Index**  **Mean ± SD** | 59.6  ± 17.1 | 64.6  ± 16.2 | <0.001 | | 60.4  ± 16.6 | 60.1  ± 17.8 | 0.253 | 0.017 |
| **Male**  **n= (%)** | 4,667  (48.7) | 13,849  (54.8) | <0.001 | | 4,523  (49.7) | 4,513  (49.5) | 0.882 | 0.002 |
| **Cardiovascular co-morbidities n= (%)** | | | | | | | |  |
| **Hypertension** | 6,904  (72.0) | 21,265  (84.2) | <0.001 | | 6,703  (73.6) | 6,709  (73.7) | 0.920 | 0.001 |
| **Ischaemic heart disease** | 2,915  (30.4) | 11,709  (46.4) | <0.001 | | 2,879  (31.6) | 2,877  (31.6) | 0.975 | <0.001 |
| **Heart failure** | 2,016  (21.0) | 10,033  (39.7) | <0.001 | | 2,008  (22.0) | 2,025  (22.2) | 0.762 | 0.004 |
| **Diabetes mellitus** | 5,202  (54.2) | 16,149  (64.0) | <0.001 | | 4,976  (54.6) | 4,969  (54.6) | 0.917 | 0.002 |
| **Smoking** | 1,334  (13.9) | 3,796  (15.0) | 0.008 | | 1,264  (13.9) | 1,331  (14.6) | 0.156 | 0.021 |
| **Cardiovascular medication n= (%)** | | | | | | | |  |
| **Beta blockers** | 4,654  (48.5) | 16,740  (66.3) | <0.001 | | 4,614  (50.7) | 4,762  (52.3) | 0.028 | 0.033 |
| **Antilipemic agents** | 5,123  (53.4) | 15,274  (60.5) | <0.001 | | 4,892  (53.7) | 5,011  (55.0) | 0.077 | 0.026 |
| **Ace inhibitors** | 3,818  (39.8) | 10,983  (43.5) | <0.001 | | 3,644  (40.0) | 3,684  (40.4) | 0.546 | 0.009 |
| **Angiotensin II inhibitor** | 2,817  (29.4) | 8,106  (32.1) | <0.001 | | 2,705  (29.7) | 2,759  (30.3) | 0.383 | 0.013 |
| **Aspirin** | 4,127  (43.0) | 13,482  (53.4) | <0.001 | | 3,997  (43.9) | 4,097  (45.0) | 0.136 | 0.022 |
| **Clopidogrel** | 1,050  (10.9) | 4,600  (18.2) | <0.001 | | 1,044  (11.5) | 1,088  (11.9) | 0.311 | 0.015 |
| **Diuretics** | 5,137  (53.6) | 16,851  (66.7) | <0.001 | | 4,994  (54.8) | 5,118  (56.2) | 0.065 | 0.027 |
| **Finerenone** | 10  (0.1) | 13  (0.1) | 0.087 | | 10  (0.1) | 10  (0.1) | 1 | <0.001 |
| **Eplerenone** | 53  (0.6) | 200  (0.8) | 0.019 | | 52  (0.6) | 50  (0.5) | 0.843 | 0.003 |
| **Spironolactone** | 883  (9.2) | 3,176  (12.6) | <0.001 | | 858  (9.4) | 838  (9.2) | 0.610 | 0.008 |
| **Laboratory results** | | | | | | | |  |
| **eGFR categories (ml/min/1.73m^2^)** | | | | | | | |  |
| **>90** | 4,552  (47.5) | 6,284  (24.9) | <0.001 | | 4,252  (46.7) | 2,532  (27.8) | <0.001 | 0.398 |
| **60-89** | 6,431  (67.1) | 11,690  (46.3) | <0.001 | | 6,110  (67.1) | 4,343  (47.7) | <0.001 | 0.400 |
| **30-59** | 5,416  (56.5) | 15,460  (61.2) | <0.001 | | 5,232  (57.4) | 5,243  (57.6) | 0.869 | 0.002 |
| **15-29** | 2,275  (23.7) | 12,375  (49.0) | <0.001 | | 2,216  (24.3) | 4,051  (44.5) | <0.001 | 0.434 |
| **< 15** | 1,261  (13.1) | 9,033  (35.8) | <0.001 | | 1,228  (13.5) | 3,007  (33.0) | <0.001 | 0.475 |
| **Proteinuria (Microalbumin mg/g)** | | | | | | | |  |
| **0 - 30** | 1,246  (13.0) | 2,055  (8.1) | <0.001 | | 1,110  (12.2) | 1,137  (12.5) | 0.543 | 0.009 |
| **30 - 300** | 1,332  (13.9) | 3,092  (12.2) | <0.001 | | 1,231  (13.5) | 1,266  (13.9) | 0.451 | 0.011 |
| **>300** | 837  (8.7) | 3,081  (12.2) | <0.001 | | 823  (9.0) | 825  (9.1) | 0.959 | 0.001 |
| **Cholesterol mg/dL (Mean ± SD)** | 178.7  ± 58.1 | 163.1  ± 56.7 | <0.001 | | 177.0  ± 56.7 | 175.1  ± 62.7 | 0.072 | 0.033 |
|  | | | | | | | |  |
| **IgA Nephropathy** | | | | | | | |  |
| **Sample Size (n=)** | 2,956 | 8,112 |  | | 2,812 | 2,812 |  | |
| **Age at Index**  **Mean ± SD** | 55.5  ± 17.4 | 61.1  ± 16.9 | <0.001 | | 56.1  ± 17.0 | 56.0  ± 18.2 | 0.760 | 0.008 |
| **Male**  **n= (%)** | 1,445(48.9) | 4,449 (54.8) | <0.001 | | 1,396  (49.6) | 1,387  (49.3) | 0.810 | 0.006 |
| **Cardiovascular co-morbidities n= (%)** | | | | | | | |  |
| **Hypertension** | 2,379(80.5) | 7,136 (88.0) | <0.001 | | 2,283  (81.2) | 2,294  (81.6) | 0.706 | 0.010 |
| **Ischaemic heart disease** | 821(27.8) | 3,658  (45.1) | <0.001 | | 817  (29.1) | 815  (29.0) | 0.953 | 0.002 |
| **Heart failure** | 598(20.2) | 3,250  (40.1) | <0.001 | | 595  (21.2) | 599  (21.3) | 0.896 | 0.003 |
| **Diabetes mellitus** | 1,332(45.1) | 4,332  (53.4) | <0.001 | | 1,278  (45.4) | 1,280  (45.5) | 0.957 | 0.001 |
| **Smoking** | 495(16.7) | 1,369 (16.9) | 0.871 | | 465  (16.5) | 477  (17.0) | 0.668 | 0.011 |
| **Cardiovascular medication n= (%)** | | | | | | | |  |
| **Beta blockers** | 1,603  (54.2) | 5,727  (70.6) | <0.001 | | 1,593  (56.7) | 1,642  (58.4) | 0.186 | 0.035 |
| **Antilipemic agents** | 1,656  (56.0) | 4,906  (60.5) | <0.001 | | 1,570  (55.8) | 1,615  (57.4) | 0.226 | 0.032 |
| **Ace inhibitors** | 1,298  (43.9) | 3,630  (44.7) | 0.433 | | 1,226  (43.6) | 1,235  (43.9) | 0.809 | 0.006 |
| **Angiotensin II inhibitor** | 970  (32.8) | 2,733  (33.7) | 0.387 | | 926  (32.9) | 958  (34.1) | 0.366 | 0.024 |
| **Aspirin** | 1,308  (44.2) | 4,512  (55.6) | <0.001 | | 1,272  (45.2) | 1,310  (46.6) | 0.309 | 0.027 |
| **Clopidogrel** | 284  (9.6) | 1,415  (17.4) | <0.001 | | 282  (10.0) | 290  (10.3) | 0.724 | 0.009 |
| **Diuretics** | 1,770  (59.9) | 5,613  (69.2) | <0.001 | | 1,702  (60.5) | 1,752  (62.3) | 0.171 | 0.037 |
| **Finerenone** | 10  (0.3) | 10  (0.1) | 0.018 | | 10  (0.4) | 0  (0) | 0.002 | 0.084 |
| **Eplerenone** | 17  (0.6) | 71  (0.9) | 0.116 | | 17  (0.6) | 25  (0.9) | 0.215 | 0.033 |
| **Spironolactone** | 284  (9.6) | 982  (12.1) | <0.001 | | 274  (9.7) | 306  (10.9) | 0.161 | 0.037 |
| **Laboratory results** | | | | | | | |  |
| **eGFR categories (ml/min/1.73m^2^)** | | | | | | | | |
| **>90** | 1,358  (45.9) | 2,064  (25.4) | <0.001 | | 1,289  (45.8) | 837  (29.8) | <0.001 | 0.336 |
| **60-89** | 2,039  (69.0) | 3,931  (48.5) | <0.001 | | 1,949  (69.3) | 1,440  (51.2) | <0.001 | 0.376 |
| **30-59** | 1,876  (63.5) | 5,325  (65.6) | 0.033 | | 1,802  (64.1) | 1,813  (64.5) | 0.760 | 0.008 |
| **15-29** | 996  (33.7) | 4,614  (56.9) | <0.001 | | 958  (34.1) | 1,503  (53.4) | <0.001 | 0.398 |
| **< 15** | 583  (19.7) | 3,741  (46.1) | <0.001 | | 566  (20.1) | 1,211  (43.1) | <0.001 | 0.509 |
| **Proteinuria (Microalbumin mg/g)** | | | | | | | |  |
| **0 - 30** | 307  (10.4) | 449  (5.5) | <0.001 | | 250  (8.9) | 254  (9.0) | 0.852 | 0.005 |
| **30 - 300** | 331  (11.2) | 794  (9.8) | 0.030 | | 300  (10.7) | 320  (11.4) | 0.394 | 0.023 |
| **>300** | 246  (8.3) | 895  (11.0) | <0.001 | | 241  (8.6) | 262  (9.3) | 0.326 | 0.026 |
| **Cholesterol mg/dL (Mean ± SD)** | 181.3  ± 60.4 | 166.2  ± 56.6 | <0.001 | | 180.2  ± 60.1 | 177.4  ± 61.5 | 0.146 | 0.046 |
|  | | | | | | | |  |
| **Membranous Nephropathy** | | | | | | | |  |
| **Sample Size (n=)** | 2,753 | 7,563 |  | | 2,618 | 2,618 |  | |
| **Age at Index**  **Mean ± SD** | 55.5  ± 17.4 | 61.2  ± 16.9 | <0.001 | | 56.1  ± 17.2 | 55.5  ± 18.7 | 0.247 | 0.032 |
| **Male**  **n= (%)** | 1,319  (47.9) | 4,091  (54.1) | <0.001 | | 1,276  (48.7) | 1,290  (49.3) | 0.699 | 0.011 |
| **Cardiovascular co-morbidities n= (%)** | | | | | | | |  |
| **Hypertension** | 2,225  (80.8) | 6,703  (88.6) | <0.001 | | 2,135  (81.6) | 2,131  (81.4) | 0.887 | 0.004 |
| **Ischaemic heart disease** | 769  (27.9) | 3,481  (46.0) | <0.001 | | 766  (29.3) | 759  (29.0) | 0.831 | 0.006 |
| **Heart failure** | 564  (20.5) | 3,084  (40.8) | <0.001 | | 563  (21.5) | 554  (21.2) | 0.761 | 0.008 |
| **Diabetes mellitus** | 1,243  (45.2) | 4,112  (54.4) | <0.001 | | 1,188  (45.4) | 1,189  (45.4) | 0.978 | 0.001 |
| **Smoking** | 471  (17.1) | 1,305  (17.3) | 0.862 | | 443  (16.9) | 439  (16.8) | 0.883 | 0.004 |
| **Cardiovascular medication n= (%)** | | | | | | | |  |
| **Beta blockers** | 1,504  (54.6) | 5,390  (71.3) | <0.001 | | 1,488  (56.8) | 1,528  (58.4) | 0.263 | 0.031 |
| **Antilipemic agents** | 1,557  (56.6) | 4,649  (61.5) | <0.001 | | 1,470  (56.1) | 1,482  (56.6) | 0.738 | 0.009 |
| **Ace inhibitors** | 1,238  (45.0) | 3,453  (45.7) | 0.535 | | 1,168  (44.6) | 1,153  (44.0) | 0.676 | 0.012 |
| **Angiotensin II inhibitor** | 891  (32.4) | 2,567  (33.9) | 0.133 | | 841  (32.1) | 832  (31.8) | 0.790 | 0.007 |
| **Aspirin** | 1,250  (45.4) | 4,299  (56.8) | <0.001 | | 1,215  (46.4) | 1,203  (46.0) | 0.739 | 0.009 |
| **Clopidogrel** | 265  (9.6) | 1,366  (18.1) | <0.001 | | 263  (10.0) | 257  (9.8) | 0.782 | 0.008 |
| **Diuretics** | 1,682  (61.1) | 5,295  (70.0) | <0.001 | | 1,613  (61.6) | 1,605  (61.3) | 0.820 | 0.006 |
| **Finerenone** | 10  (0.4) | 10  (0.1) | 0.018 | | 10  (0.4) | 10  (0.4) | 1 | <0.001 |
| **Eplerenone** | 16  (0.6) | 69  (0.9) | 0.100 | | 16  (0.6) | 14  (0.5) | 0.714 | 0.010 |
| **Spironolactone** | 272  (9.9) | 936  (12.4) | <0.001 | | 262  (10.0) | 238  (9.1) | 0.259 | 0.031 |
| **Laboratory results** | | | | | | | |  |
| **eGFR categories (ml/min/1.73m^2^)** | | | | | | | |  |
| **>90** | 1,276  (46.3) | 1,943  (25.7) | <0.001 | | 1,205  (46.0) | 721  (27.5) | <0.001 | 0.391 |
| **60-89** | 1,919  (69.7) | 3,728  (49.3) | <0.001 | | 1,829  (69.9) | 1,275  (48.7) | <0.001 | 0.441 |
| **30-59** | 1,758  (63.9) | 5,059  (66.9) | 0.004 | | 1,693  (64.7) | 1,641  (62.7) | 0.135 | 0.041 |
| **15-29** | 943  (34.3) | 4,358  (57.6) | <0.001 | | 915  (35.0) | 1,367  (52.2) | <0.001 | 0.354 |
| **< 15** | 540  (19.6) | 3,498  (46.3) | <0.001 | | 527  (20.1) | 1,121  (42.8) | <0.001 | 0.504 |
| **Proteinuria (Microalbumin mg/g)** | | | | | | | |  |
| **0 - 30** | 304  (11.0) | 442  (5.8) | <0.001 | | 243  (9.3) | 234  (8.9) | 0.666 | 0.012 |
| **30 - 300** | 324  (11.8) | 780  (10.3) | 0.034 | | 289  (11.0) | 285  (10.9) | 0.860 | 0.005 |
| **>300** | 237  (8.6) | 866  (11.5) | <0.001 | | 230  (8.8) | 224  (8.6) | 0.768 | 0.008 |
| **Cholesterol mg/dL (Mean ± SD)** | 181.5  ± 61.1 | 166.2  ± 56.8 | <0.001 | | 180.7  ± 61.2 | 178.1  ± 60.5 | 0.197 | 0.043 |
|  | | | | | | | |  |
| **Focal segmental Glomerulosclerosis** | | | | | | | |  |
| **Sample Size (n=)** | 2,946 | 8,082 |  | | 2,810 | 2,810 |  | |
| **Age at Index**  **Mean ± SD** | 55.8  ± 17.4 | 61.5  ± 17.0 | <0.001 | | 56.4  ± 17.1 | 56.0  ± 18.8 | 0.454 | 0.020 |
| **Male**  **n= (%)** | 1,413  (48.0) | 4,342  (53.7) | <0.001 | | 1,362  (48.5) | 1,399  (49.8) | 0.324 | 0.026 |
| **Cardiovascular co-morbidities n= (%)** | | | | | | | |  |
| **Hypertension** | 2,378  (80.7) | 7,159  (88.6) | <0.001 | | 2,290  (81.5) | 2,303  (82.0) | 0.654 | 0.012 |
| **Ischaemic heart disease** | 823  (27.9) | 3,713  (45.9) | <0.001 | | 816  (29.0) | 826  (29.4) | 0.769 | 0.008 |
| **Heart failure** | 608  (20.6) | 3,330  (41.2) | <0.001 | | 606  (21.6) | 639  (22.7) | 0.289 | 0.028 |
| **Diabetes mellitus** | 1,303  (44.2) | 4,378  (54.2) | <0.001 | | 1,247  (44.4) | 1,280  (45.6) | 0.376 | 0.024 |
| **Smoking** | 513  (17.4) | 1,397  (17.3) | 0.875 | | 483  (17.2) | 504  (17.9) | 0.462 | 0.020 |
| **Cardiovascular medication n= (%)** | | | | | | | |  |
| **Beta blockers** | 1,611  (54.7) | 5,784  (71.6) | <0.001 | | 1,595  (56.8) | 1,629  (58.0) | 0.359 | 0.024 |
| **Antilipemic agents** | 1,646(55.9) | 4,983 (61.7) | <0.001 | | 1,562  (55.6) | 1,594  (56.7) | 0.390 | 0.023 |
| **Ace inhibitors** | 1,316(44.7) | 3,673 (45.4) | 0.469 | | 1,240  (44.1) | 1,260  (44.8) | 0.591 | 0.014 |
| **Angiotensin II inhibitor** | 939(31.9) | 2,725 (33.7) | 0.069 | | 896  (31.9) | 882  (31.4) | 0.688 | 0.011 |
| **Aspirin** | 1,350(45.8) | 4,632 (57.3) | <0.001 | | 1,313  (46.7) | 1,342  (47.8) | 0.438 | 0.021 |
| **Clopidogrel** | 283(9.6) | 1,455  (18.0) | <0.001 | | 283  (10.1) | 286  (10.2) | 0.894 | 0.004 |
| **Diuretics** | 1,809(61.4) | 5,677 (70.2) | <0.001 | | 1,744  (62.1) | 1,760  (62.6) | 0.660 | 0.012 |
| **Finerenone** | 10(0.3) | 10 (0.1) | 0.018 | | 10  (0.4) | 10  (0.4) | 1 | <0.001 |
| **Eplerenone** | 17(0.6) | 72 (0.9) | 0.103 | | 17  (0.6) | 20  (0.7) | 0.621 | 0.013 |
| **Spironolactone** | 295(10.0) | 1,013  (12.5) | <0.001 | | 286  (10.2) | 314  (11.2) | 0.226 | 0.032 |
| **Laboratory results** | | | | | | | |  |
| **eGFR categories (ml/min/1.73m^2^)** | | | | | | | |  |
| **>90** | 1,391  (47.2) | 2,092  (25.9) | <0.001 | | 1,319  (46.9) | 819  (29.1) | <0.001 | 0.373 |
| **60-89** | 2,071  (70.3) | 4,015 (49.7) | <0.001 | | 1,980  (70.5) | 1,412  (50.2) | <0.001 | 0.422 |
| **30-59** | 1,911  (64.9) | 5,445  (67.4) | 0.014 | | 1,842  (65.6) | 1,798  (64.0) | 0.219 | 0.033 |
| **15-29** | 1,047  (35.5) | 4,696  (58.1) | <0.001 | | 1,016  (36.2) | 1,505  (53.6) | <0.001 | 0.355 |
| **< 15** | 600  (20.4) | 3,731  (46.2) | <0.001 | | 585  (20.8) | 1,192  (42.4) | <0.001 | 0.478 |
| **Proteinuria (Microalbumin mg/g)** | | | | | | | |  |
| **0 - 30** | 324  (11.0) | 473  (5.9) | <0.001 | | 263  (9.4) | 287  (10.2) | 0.281 | 0.029 |
| **30 - 300** | 336  (11.4) | 819  (10.1) | 0.054 | | 308  (11.0) | 326  (11.6) | 0.448 | 0.020 |
| **>300** | 239  (8.1) | 886  (11.0) | <0.001 | | 233  (8.3) | 233  (8.3) | 1 | <0.001 |
| **Cholesterol mg/dL (Mean ± SD)** | 180.8  ± 59.9 | 165.5  ± 56.1 | <0.001 | | 179.9  ± 59.0 | 176.8  ± 59.9 | 0.102 | 0.052 |
|  | | | | | | | |  |
| **Minimal Change Disease** | | | | | | | |  |
| **Sample Size (n=)** | 3,174 | 8,435 |  | | 3,016 | 3,016 |  |  |
| **Age at Index**  **Mean ± SD** | 56.0 ± 17.7 | 61.7 ± 17.1 | <0.001 | | 56.8  ± 17.3 | 56.2  ± 19.2 | 0.206 | 0.033 |
| **Male**  **n= (%)** | 1,526  (48.1) | 4,543  (53.9) | <0.001 | | 1,461  (48.4) | 1,494  (49.5) | 0.395 | 0.022 |
| **Cardiovascular co-morbidities n= (%)** | | | | | | | |  |
| **Hypertension** | 2,519  (79.4) | 7,418  (87.9) | <0.001 | | 2,430  (80.6) | 2,422  (80.3) | 0.795 | 0.007 |
| **Ischaemic heart disease** | 896  (28.2) | 3,877  (46.0) | <0.001 | | 89  1(29.5) | 920  (30.5) | 0.415 | 0.021 |
| **Heart failure** | 645  (20.3) | 3,454  (40.9) | <0.001 | | 644  (21.4) | 648  (21.5) | 0.900 | 0.003 |
| **Diabetes mellitus** | #1,411  (44.5) | 4,565  (54.1) | <0.001 | | 1,362  (45.2) | 1,385  (45.9) | 0.552 | 0.015 |
| **Smoking** | 534  (16.8) | 1,436  (17.0) | 0.798 | | 505  (16.7) | 500  (16.6) | 0.863 | 0.004 |
| **Cardiovascular medication n= (%)** | | | | | | | |  |
| **Beta blockers** | 1,696  (53.4) | 5,978  (70.9) | <0.001 | | 1,681  (55.7) | 1,695  (56.2) | 0.717 | 0.009 |
| **Antilipemic agents** | 1,745  (55.0) | 5,180  (61.4) | <0.001 | | 1,664  (55.2) | 1,655  (54.9) | 0.816 | 0.006 |
| **Ace inhibitors** | 1,368  (43.1) | 3,801  (45.1) | 0.058 | | 1,295  (42.9) | 1,311  (43.5) | 0.677 | 0.011 |
| **Angiotensin II inhibitor** | 998  (31.4) | 2,849  (33.8) | 0.017 | | 950  (31.5) | 937  (31.1) | 0.718 | 0.009 |
| **Aspirin** | 1,431  (45.1) | 4,784  (56.7) | <0.001 | | 1,40  5(46.6) | 1,412  (46.8) | 0.857 | 0.005 |
| **Clopidogrel** | 12  (9.8) | 1,519  (18.0) | <0.001 | | 311  (10.3) | 319  (10.6) | 0.736 | 0.009 |
| **Diuretics** | 1,904  (60.0) | 5,906  (70.0) | <0.001 | | 1,831  (60.7) | 1,836  (60.9) | 0.895 | 0.003 |
| **Finerenone** | 10  (0.3) | 10  (0.1) | 0.023 | | 10  (0.3) | 10  (0.3) | 1 | <0.001 |
| **Eplerenone** | 18  (0.6) | 75  (0.9) | 0.083 | | 18  (0.6) | 22  (0.7) | 0.526 | 0.016 |
| **Spironolactone** | 314  (9.9) | 1,057  (12.5) | <0.001 | | 306  (10.1) | 324  (10.7) | 0.449 | 0.020 |
| **Laboratory results** | | | | | | | |  |
| **eGFR categories (ml/min/1.73m^2^)** | | | | | | | |  |
| **>90** | 1,508  (47.5) | 2,257  (26.8) | <0.001 | | 1,424  (47.2) | 858  (28.4) | <0.001 | 0.394 |
| **60-89** | ,225  (70.1) | 4,265  (50.6) | <0.001 | | 2,124  (70.4) | 1,493  (49.5) | <0.001 | 0.437 |
| **30-59** | 2,013  (63.4) | 5,687  (67.4) | <0.001 | | 1,943  (64.4) | 1,885  (62.5) | 0.121 | 0.040 |
| **15-29** | 1,071  (33.7) | 4,811  (57.0) | <0.001 | | 1,040  (34.5) | 1,544  (51.2) | <0.001 | 0.343 |
| **< 15** | 00  (18.9) | 3,772  (44.7) | <0.001 | | 589  (19.5) | 1,206  (40.0) | <0.001 | 0.459 |
| **Proteinuria (Microalbumin)** |  | | | | | | |  |
| **0 - 30 mg/g** | 340  (10.7) | 488  (5.8) | <0.001 | | 283  (9.4) | 282  (9.4) | 0.965 | 0.001 |
| **30 - 300 mg/g** | 359  (11.3) | 851  (10.1) | 0.055 | | 332  (11.0) | 332  (11.0) | 1 | <0.001 |
| **>300mg/g** | 259  (8.2) | 943  (11.2) | <0.001 | | 256  (8.5) | 255  (8.5) | 0.963 | 0.001 |
| **Cholesterol mg/dL (Mean ± SD)** | 181.3  ± 61.8 | 165.4  ± 56.3 | <0.001 | | 179.6  ± 59.8 | 176.3  ± 60.6 | 0.076 | 0.055 |

Table showing the demographics and cardiovascular risk factors for all cause GN and primary GN sub-type cohorts for NTproBNP. Results prior to and following propensity score matching (PSM) are included. All statistical analysis was performed using the online TriNetX platform. 1:1PSM using logistic regression. The cohorts were matched for age, sex, and comorbidities influencing adverse CV outcomes (hypertension, heart failure diabetes mellitus and smoking status) at baseline. A P<0.05 was accepted as statistically significant.

**Appendix Table 4.** Survival analysis for each GN sub-group; Number of patients with each MACE outcome, Hazard ratio and 95% confidence interval for Troponin I at a threshold of 18ng/L.

|  | **Outcome** | **Troponin I** | | | | | | | | |
| --- | --- | --- | --- | --- | --- | --- | --- | --- | --- | --- |
|  |  | **<18 ng/L** | | **≥18 ng/L** | | | | | | |
| **All Cause GN** |  | **Cohort**  **N=** | **Outcome**  **N=** | | **Cohort**  **N=** | **Outcome**  **N=** | **HR** | **95% CI** | **χ^2^** | **P-Value** |
|  | **MACE** | 7,923 | 3,000 | | 5,702 | 3,222 | 1.79 | (1.70,1.88) | 63.379 | <0.0001 |
|  | **IHD** | 11,287 | 2,355 | | 9,559 | 2,843 | 1.62 | (1.53,1.71) | 48.582 | <0.0001 |
|  | **Angina** | 15,797 | 795 | | 15,861 | 1,018 | 1.33 | (1.22,1.46) | 20.259 | <0.0001 |
|  | **Acute MI** | 16,107 | 1,355 | | 14,378 | 2,040 | 1.80 | (1.68,1.93) | 44.044 | <0.0001 |
|  | **Heart Failure** | 12,331 | 2,228 | | 10,866 | 3,148 | 1.81 | (1.71,1.91) | 41.632 | <0.0001 |
|  | **Atrial fibrillation and flutter** | 14,747 | 1,318 | | 14,077 | 1,776 | 1.48 | (1.38,1.59) | 3.834 | 0.050 |
|  | **Cerebral Infarction** | 15,796 | 939 | | 15,825 | 1,155 | 1.27 | (1.17,1.38) | 9.236 | 0.002 |
|  | **Deceased** | 17,352 | 3,886 | | 17,250 | 5,656 | 1.53 | (1.47,1.59) | 54.427 | <0.0001 |
| **IgA Nephropathy** | **MACE** | 3,190 | 1,126 | | 2,298 | 1,199 | 1.75 | (1.61,1.90) | 22.623 | <0.0001 |
|  | **IHD** | 4,363 | 827 | | 3,735 | 1,050 | 1.68 | (1.54,1.85) | 17.801 | <0.0001 |
|  | **Angina** | 5,897 | 263 | | 5,905 | 337 | 1.34 | (1.14,1.57) | 13.642 | <0.0001 |
|  | **Acute MI** | 5,908 | 453 | | 5,257 | 705 | 1.87 | (1.66,2.10) | 15.654 | <0.0001 |
|  | **Heart Failure** | 4,711 | 733 | | 4,228 | 1,100 | 1.87 | (1.70,2.05) | 18.873 | <0.0001 |
|  | **Atrial fibrillation and flutter** | 5,523 | 458 | | 5,192 | 633 | 1.54 | (1.36,1.73) | 1.181 | 0.277 |
|  | **Cerebral Infarction** | 5,857 | 317 | | 5,815 | 387 | 1.27 | (1.10,1.47) | 6.922 | 0.009 |
|  | **Deceased** | 6,345 | 1,225 | | 6,310 | 1,808 | 1.54 | (1.43,1.66) | 8.715 | 0.003 |
| **Membranous Nephropathy (MN)** | **MACE** | 2,923 | 1,058 | | 2,179 | 1,173 | 1.79 | (1.64,1.94) | 18.235 | <0.0001 |
|  | **IHD** | 4,034 | 778 | | 3,488 | 1,020 | 1.74 | (1.59,1.91) | 18.266 | <0.0001 |
|  | **Angina** | 5,485 | 254 | | 5,470 | 331 | 1.37 | (1.16,1.61) | 9.298 | 0.002 |
|  | **Acute MI** | 5,496 | 431 | | 4,915 | 683 | 1.89 | (1.67,2.13) | 13.162 | <0.0001 |
|  | **Heart Failure** | 4,369 | 692 | | 3,929 | 1,054 | 1.90 | (1.73,2.09) | 13.441 | <0.0001 |
|  | **Atrial fibrillation and flutter** | 5,148 | 439 | | 4,849 | 611 | 1.54 | (1.36,1.74) | 0.676 | 0.411 |
|  | **Cerebral Infarction** | 5,445 | 309 | | 5,408 | 356 | 1.20 | (1.03,1.39) | 4.221 | 0.040 |
|  | **Deceased** | 5,920 | 1,164 | | 5,890 | 1,720 | 1.54 | (1.43,1.66) | 4.517 | 0.034 |
| **Focal**  **Segmental**  **Glomerulo-sclerosis**  **(FSGS)** | **MACE** | 3,129 | 1,143 | | 2,282 | 1,213 | 1.71 | (1.58,1.86) | 22.427 | <0.0001 |
|  | **IHD** | 4,310 | 852 | | 3,675 | 1,033 | 1.62 | (1.48,1.77) | 25.936 | <0.0001 |
|  | **Angina** | 5,865 | 270 | | 5,865 | 344 | 1.34 | (1.14,1.57) | 11.464 | 0.001 |
|  | **Acute MI** | 5,886 | 480 | | 5,228 | 725 | 1.83 | (1.63,2.05) | 16.059 | <0.0001 |
|  | **Heart Failure** | 4,675 | 754 | | 4,208 | 1,119 | 1.84 | (1.67,2.01) | 15.429 | <0.0001 |
|  | **Atrial fibrillation and flutter** | 5,482 | 463 | | 5,165 | 652 | 1.56 | (1.39,1.76) | 1.550 | 0.213 |
|  | **Cerebral Infarction** | 5,833 | 318 | | 5,801 | 371 | 1.22 | (1.05,1.41) | 2.674 | 0.102 |
|  | **Deceased** | 1.537 | 1,265 | | 6,297 | 1,851 | 1.54 | (1.43,1.65) | 9.613 | 0.002 |
| **Minimal change disease**  **(MCD)** | **MACE** | 3,225 | 1,162 | | 2,325 | 1,225 | 1.71 | (1.58,1.86) | 16.520 | <0.0001 |
|  | **IHD** | 4,445 | 857 | | 3,757 | 1,035 | 1.62 | (1.48,1.77) | 24.148 | <0.0001 |
|  | **Angina** | 6,037 | 279 | | 6,009 | 353 | 1.33 | (1.14,1.56) | 17.861 | <0.0001 |
|  | **Acute MI** | 6,068 | 482 | | 5,360 | 745 | 1.87 | (1.67,2.10) | 12.935 | <0.0001 |
|  | **Heart Failure** | 4,832 | 744 | | 4,320 | 1,088 | 1.83 | (1.67,2.01) | 13.693 | <0.0001 |
|  | **Atrial fibrillation and flutter** | 5,633 | 478 | | 5,314 | 663 | 1.54 | (1.37,1.73) | 0.504 | 0.478 |
|  | **Cerebral Infarction** | 5,980 | 323 | | 5,943 | 378 | 1.21 | (1.05,1.41) | 3.532 | 0.060 |
|  | **Deceased** | 6,515 | 1,306 | | 6,464 | 1,916 | 1.54 | (1.44,1.65) | 6.590 | 0.010 |
| *Table showing the survival analysis for Troponin I all cause GN and primary GN sub-type cohort. Survival analysis estimates the probability of an outcome at a respective time over 5 years follow-up from the index event. Statistical analysis was performed using the' Analytics' functionality on TriNetX which used the R Survival package v3.2-3 for its analysis. Results are reported as hazard ratio (HR),95% Confidence interval (CI) and log-rank tests(χ^2^). A p-value <0.05 was accepted as the level of statistical significance.* | | | | | | | | | | |

**Appendix Table 5.** Survival analysis for each GN sub-group; Number of patients with each MACE outcome, Hazard ratio and 95% confidence interval for NTproBNP at a threshold of 400 pg/ml.

|  |  | **NT-proBNP** | | | | | | | |
| --- | --- | --- | --- | --- | --- | --- | --- | --- | --- |
|  |  | **< 400 pg/ml** | | **≥ 400 pg/ml** | | | | | |
| **All Cause GN** |  | **Cohort**  **N=** | **Outcome**  **N=** | **Cohort**  **N=** | **Outcome**  **N=** | **HR** | **95% CI** | **χ^2^** | **P-Value** |
|  | **MACE** | 3,942 | 1,337 | 3,174 | 1,686 | 1.99 | (1.86,2.14) | 36.615 | <0.0001 |
|  | **IHD** | 5,861 | 933 | 5,461 | 1,369 | 1.83 | (1.69,1.99) | 21.460 | <0.0001 |
|  | **Angina** | 8,007 | 358 | 8,264 | 441 | 1.29 | (1.13,1.49) | 0.062 | 0.803 |
|  | **Acute MI** | 8,142 | 526 | 7,700 | 844 | 1.91 | (1.71,2.13) | 20.311 | <0.0001 |
|  | **Heart Failure** | 6,268 | 982 | 5,400 | 1,559 | 2.26 | (2.08,2.44) | 62.529 | <0.0001 |
|  | **Atrial fibrillation and flutter** | 8,144 | 567 | 7,250 | 887 | 1.96 | (1.76,2.17) | 20.139 | <0.0001 |
|  | **Cerebral Infarction** | 8,217 | 364 | 8,170 | 494 | 1.49 | (1.30,1.70) | 6.286 | 0.012 |
|  | **Deceased** | 8,985 | 1,310 | 8,966 | 2,901 | 2.49 | (2.33,2.66) | 19.264 | <0.0001 |
| **IgA Nephropathy** | **MACE** | 1,380 | 434 | 1,073 | 508 | 1.84 | (1.62,2.09) | 15.625 | <0.0001 |
|  | **IHD** | 1,911 | 260 | 1,762 | 408 | 1.97 | (1.68,2.30) | 9.954 | 0.002 |
|  | **Angina** | 2,520 | 90 | 2,573 | 122 | 1.45 | (1.10,1.90) | 0.422 | 0.516 |
|  | **Acute MI** | 2,576 | 126 | 2,412 | 258 | 2.45 | (1.98,3.03) | 8.596 | 0.003 |
|  | **Heart Failure** | 2,105 | 269 | 1,758 | 457 | 2.46 | (2.11,2.86) | 11.791 | 0.001 |
|  | **Atrial fibrillation and flutter** | 2,502 | 165 | 2,236 | 265 | 1.98 | (1.63,2.41) | 4.336 | 0.037 |
|  | **Cerebral Infarction** | 2,571 | 103 | 2,544 | 136 | 1.46 | (1.13,1.89) | 3.410 | 0.065 |
|  | **Deceased** | 2,773 | 384 | 2,765 | 799 | 2.31 | (2.05,2.61) | 14.590 | <0.0001 |
| **Membranous Nephropathy (MN)** | **MACE** | 1,284 | 409 | 994 | 492 | 1.91 | (1.68,2.18) | 12.260 | <0.0001 |
|  | **IHD** | 1,782 | 251 | 1,639 | 362 | 1.78 | (1.52,2.09) | 11.161 | 0.001 |
|  | **Angina** | 2,343 | 84 | 2,392 | 115 | 0.21 | (1.09,1.92) | 1.562 | 0.211 |
|  | **Acute MI** | 2,403 | 122 | 2,233 | 222 | 2.18 | (1.75,2.72) | 11.120 | 0.001 |
|  | **Heart Failure** | 1,966 | 257 | 1,632 | 425 | 2.43 | (2.08,2.84) | 13.097 | <0.0001 |
|  | **Atrial fibrillation and flutter** | 2,324 | 161 | 2,113 | 249 | 1.86 | (1.53,2.27) | 2.186 | 0.139 |
|  | **Cerebral Infarction** | 2,388 | 97 | 2,376 | 131 | 1.48 | (1.14,1.92) | 1.290 | 0.256 |
|  | **Deceased** | 2,580 | 365 | 2,579 | 778 | 2.39 | (2.11,2.70) | 16.626 | <0.0001 |
| **Focal**  **Segmental**  **Glomerulo-sclerosis**  **(FSGS)** | **MACE** | 1,382 | 431 | 1,040 | 501 | 1.88 | (1.65,2.14) | 22.405 | <0.0001 |
|  | **IHD** | 1,917 | 277 | 1,769 | 392 | 1.72 | (1.48,2.01) | 9.845 | 0.002 |
|  | **Angina** | 2,523 | 97 | 2,559 | 129 | 1.42 | (1.09,1.85) | 0.330 | 0.566 |
|  | **Acute MI** | 2,578 | 129 | 2,433 | 245 | 2.25 | (1.82,2.79) | 16.043 | <0.0001 |
|  | **Heart Failure** | 2,108 | 272 | 1,713 | 435 | 2.34 | (2.01,2.72) | 11.745 | 0.001 |
|  | **Atrial fibrillation and flutter** | 2,486 | 167 | 2,239 | 258 | 1.87 | (1.54,2.27) | 4.325 | 0.038 |
|  | **Cerebral Infarction** | 2,553 | 104 | 2,550 | 138 | 1.45 | (1.13,1.87) | 0.000 | 0.989 |
|  | **Deceased** | 2,772 | 395 | 2,773 | 847 | 2.4 | (2.13,2.70) | 24.549 | <0.0001 |
| **Minimal change disease**  **(MCD)** | **MACE** | 1,471 | 467 | 1,114 | 518 | 1.77 | (1.56,2.00) | 22.444 | <0.0001 |
|  | **IHD** | 2,046 | 291 | 1,856 | 385 | 1.65 | (1.42,1.92) | 13.209 | <0.0001 |
|  | **Angina** | 2,703 | 98 | 2,746 | 128 | 1.41 | (1.08,1.84) | 1.810 | 0.178 |
|  | **Acute MI** | 2,764 | 135 | 2,581 | 253 | 2.25 | (1.83,2.78) | 17.774 | <0.0001 |
|  | **Heart Failure** | 2,262 | 294 | 1,866 | 461 | 2.31 | (1.99,2.67) | 15.132 | <0.0001 |
|  | **Atrial fibrillation and flutter** | 2,673 | 183 | 2,411 | 272 | 1.82 | (1.51,2.20) | 3.446 | 0.063 |
|  | **Cerebral Infarction** | 2,743 | 108 | 2,740 | 154 | 1.57 | (1.23,2.01) | 0.348 | 0.555 |
|  | **Deceased** | 2,970 | 416 | 2,968 | 893 | 2.41 | (2.14,2.71) | 26.844 | <0.0001 |

Table showing the survival analysis for NT-proBNP all cause GN and primary GN sub-type cohort. Survival analysis estimates the probability of an outcome at a respective time over 5 years follow-up from the index event. Statistical analysis was performed using the' Analytics' functionality on TriNetX which used the R Survival package v3.2-3 for its analysis. Results are reported as hazard ratio (HR),95% Confidence interval (CI) and log-rank tests(χ^2^). A p-value <0.05 was accepted as the level of statistical significance.

**Appendix Table 6.** Demographics of sensitivity analysis Troponin I and NTproBNP adjusted for baseline kidney function profile pre and post propensity score matching.

| **TROPONIN I** | | | | | | | |
| --- | --- | --- | --- | --- | --- | --- | --- |
|  | **Initial Population (Pre-PSM)** | | | **Propensity Score Matched (PSM) Population** | | | |
|  | **<18ng/L** | **≥ 18 ng/L** | **P-value** | **<18ng/L** | **≥ 18 ng/L** | **P-value** | **SMD** |
| **Sample Size (n=)** | 18,091 | 30,450 |  | 16,911 | 16,911 |  |  |
| **Age at Index**  **Mean ± SD** | 58.8  ± 17.1 | 61.9  ± 16.3 | <0.001 | 59.5  ± 16.8 | 59.7  ± 17.1 | 0.212 | 0.014 |
| **Male**  **n= (%)** | 8,453  (46.7) | 16,741  (56.2) | <0.001 | 8,262  (48.9) | 8,221  (48.6) | 0.656 | 0.005 |
| **Cardiovascular co-morbidities n= (%)** | | | | | | |  |
| **Hypertension** | 14,824  (81.9) | 24,472  (82.2) | 0.449 | 13,858  (81.9) | 13,798  (81.6) | 0.398 | 0.009 |
| **Ischaemic heart disease** | 5,507  (30.4) | 11,583  (38.9) | <0.001 | 5,399  (31.9) | 5,400  (31.9) | 0.991 | <0.001 |
| **Heart failure** | 4,166  (23.0) | 9,369  (31.5) | <0.001 | 4,115  (24.3) | 4,149  (24.5) | 0.667 | 0.005 |
| **Diabetes mellitus** | 10,122  (56.0) | 17,622  (59.2) | <0.001 | 9,529  (56.3) | 9,570  (56.6) | 0.653 | 0.005 |
| **Smoking** | 3,066  (16.9) | 4,485  (15.1) | <0.001 | 2,809  (16.6) | 2,792  (16.5) | 0.804 | 0.003 |
| **RACE n= (%)** | | | | | | |  |
| **White** | 9,275  (51.3) | 14,782  (49.7) | 0.001 | 8,685  (51.4) | 8,639  (51.1) | 0.617 | 0.005 |
| **American Indian or Alaska Native** | 120  (0.7) | 190  (0.6) | 0.741 | 107  (0.6) | 114  (0.7) | 0.637 | 0.005 |
| **Native Hawaiian or Other Pacific Islander** | 144  (0.8) | 578  (1.9) | <0.001 | 144  (0.9) | 137  (0.8) | 0.675 | 0.005 |
| **Black or African American** | 4,615  (25.5) | 7,013  (23.6) | <0.001 | 4,266  (25.2) | 4,336  (25.6) | 0.382 | 0.010 |
| **Asian** | 619  (3.4) | 1,324  (4.4) | <0.001 | 603  (3.6) | 601  (3.6) | 0.953 | 0.001 |
| **Unknown Race** | 2,585  (14.3) | 4,699  (15.8) | <0.001 | 2,438  (14.4) | 2,401  (14.2) | 0.566 | 0.006 |
| **Cardiovascular medication n= (%)** | | | | | | |  |
| **Beta blockers** | 10,515  (58.1) | 17,867  (60.0) | <0.001 | 9,912  (58.6) | 9,928  (58.7) | 0.860 | 0.002 |
| **Antilipemic agents** | 10,034  (55.5) | 15,854  (53.3) | <0.001 | 9,298  (55.0) | 9,348  (55.3) | 0.585 | 0.006 |
| **Ace inhibitors** | 7,883  (43.6) | 12,164  (40.9) | <0.001 | 7,280  (43.0) | 7,345  (43.4) | 0.476 | 0.008 |
| **Angiotensin II inhibitor** | 5,429  (30.0) | 7,984  (26.8) | <0.001 | 4,981  (29.5) | 4,908  (29.0) | 0.383 | 0.009 |
| **Aspirin** | 7,718  (42.7) | 12,623  (42.4) | 0.585 | 7,203  42.6) | 7,236  (42.8) | 0.717 | 0.004 |
| **Clopidogrel** | 1,978  (10.9) | 3,849  (12.9) | <0.001 | 1,931  (11.4) | 1,933  (11.4) | 0.973 | <0.001 |
| **Diuretics** | 10,583  (58.5) | 17,744  (59.6) | 0.016 | 9,945  (58.8) | 9,940  (58.8) | 0.956 | 0.001 |
| **Finerenone** | 10  (0.1) | 10  (0.0) | 0.260 | 10  (0.1) | 10  (0.1) | 1 | <0.001 |
| **Eplerenone** | 77  (0.4) | 139  (0.5) | 0.513 | 75  (0.4) | 84  (0.5) | 0.474 | 0.008 |
| **Spironolactone** | 1,720  (9.5) | 2,799  (9.4) | 0.706 | 1,619  (9.6) | 1,627  (9.6) | 0.883 | 0.002 |
| **LABORATORY** | | | | | | |  |
| **eGFR***  **Mean ± SD** | 50.3  ± 34.1 | 36.0  ± 29.6 | <0.001 | 48.5  ± 33.5 | 42.5  ± 31.9 | <0.001 | 0.185 |
| **eGFR categories (ml/min/1.73m^2^)** | | | | | | | |
| **>90** | 6,020  (33.3) | 6,597  (22.2) | <0.001 | 5,169  (30.6) | 5,148  (30.4) | 0.804 | 0.003 |
| **60-89** | 9,987  (55.2) | 12,432  (41.8) | <0.001 | 8,953  (52.9) | 8,980  (53.1) | 0.769 | 0.003 |
| **30-59** | 10,887  (60.2) | 17,083  (57.4) | <0.001 | 10,181  (60.2) | 10,258  (60.7) | 0.392 | 0.009 |
| **15-29** | 7,031  (38.9) | 13,911  (46.7) | <0.001 | 6,840  (40.4) | 6,858  (40.6) | 0.842 | 0.002 |
| **< 15** | 5,636  (31.2) | 11,623  (39.0) | <0.001 | 5,496  (32.5) | 5,416  (32.0) | 0.352 | 0.010 |
| **Proteinuria (Microalbumin mg/g)** | | | | | | | |
| **0 - 30** | 1,953  (10.8) | 2,319  (7.8) | <0.001 | 1,698  (10.0) | 1,749  (10.3) | 0.359 | 0.010 |
| **30 - 300** | 2,241  (12.4) | 3,280  (11.0) | <0.001 | 2,064  (12.2) | 2,134  (12.6) | 0.248 | 0.013 |
| **>300** | 1,766  (9.8) | 3,049  (10.2) | 0.090 | 1,675  (9.9) | 1,736  (10.3) | 0.271 | 0.012 |
| **Cholesterol mg/dL (Mean ± SD)** | 172.2  ± 57.1 | 170.4  ± 61.5 | 0.008 | 171.3  ± 56.4 | 174.8  ± 63.9 | <0.001 | 0.058 |
|  | | | | | | | |
| **NTproBNP** | | | | | | | |
|  | **Initial Population (Pre-PSM)** | | | **Propensity Score Matched (PSM) Population** | | | |
|  | **NTproBNP <400 pg/ml** | **NTproBNP**  **≥400 pg/ml** | **P-value** | **NTproBNP <400 pg/ml** | **NTproBNP**  **≥400 pg/ml** | **P-value** | **SMD** |
| **Sample Size (n=)** | 9,590 | 25,251 |  | 8,365 | 8,365 |  | |
| **Age at Index**  **Mean ± SD** | 59.6  ± 17.1 | 64.6  ± 16.2 | <0.001 | 60.7  ± 16.8 | 60.7  ± 18.2 | 0.858 | 0.003 |
| **Male**  **n= (%)** | 4,667  (48.7) | 13,849  (54.8) | <0.001 | 4,253  (50.8) | 4,311  (51.5) | 0.370 | 0.014 |
| **Cardiovascular co-morbidities n= (%)** | | | | | | | |
| **Hypertension** | 6,904  (72.0) | 21,265  (84.2) | <0.001 | 6,201  (74.1) | 6,168  (73.7) | 0.561 | 0.009 |
| **Ischaemic heart disease** | 2,915  (30.4) | 11,709  (46.4) | <0.001 | 2,738  (32.7) | 2,672  (31.9) | 0.275 | 0.017 |
| **Heart failure** | 2,016  (21.0) | 10,033  (39.7) | <0.001 | 1,941  (23.2) | 1,889  (22.6) | 0.339 | 0.015 |
| **Diabetes mellitus** | 5,202  (54.2) | 16,149  (64.0) | <0.001 | 4,605  (55.1) | 4,586  (54.8) | 0.768 | 0.005 |
| **Smoking** | 1,334  (13.9) | 3,796  (15.0) | 0.008 | 1,170  (14.0) | 1,196  (14.3) | 0.564 | 0.009 |
| **RACE n= (%)** | | | | | | | |
| **White** | 4,261  (44.4) | 12,179  (48.2) | <0.001 | 3,789  (45.3) | 3,792  (45.3) | 0.963 | 0.001 |
| **American Indian or Alaska Native** | 40  (0.4) | 154  (0.6) | 0.031 | 38  (0.5) | 30  (0.4) | 0.331 | 0.015 |
| **Native Hawaiian or Other Pacific Islander** | 74  (0.8) | 289  (1.1) | 0.002 | 71  (0.8) | 66  (0.8) | 0.668 | 0.007 |
| **Black or African American** | 1,587  (16.5) | 3,886  (15.4) | 0.008 | 1,348  (16.1) | 1,381  (16.5) | 0.490 | 0.011 |
| **Asian** | 268  (2.8) | 802  (3.2) | 0.065 | 221  (2.6) | 230  (2.7) | 0.667 | 0.007 |
| **Unknown Race** | 3,167  (33.0) | 7,384  (29.2) | <0.001 | 2,725  (32.6) | 2,689  (32.1) | 0.552 | 0.009 |
| **Cardiovascular medication n= (%)** | | | | | | | |
| **Beta blockers** | 4,654  (48.5) | 16,740  (66.3) | <0.001 | 4,359  (52.1) | 4,344  (51.9) | 0.816 | 0.004 |
| **Antilipemic agents** | 5,123  (53.4) | 15,274  (60.5) | <0.001 | 4,514  (54.0) | 4,534  (54.2) | 0.756 | 0.005 |
| **Ace inhibitors** | 3,818  (39.8) | 10,983  (43.5) | <0.001 | 3,360  (40.2) | 3,379  (40.4) | 0.765 | 0.005 |
| **Angiotensin II** | 2,17  (29.4) | 8,106  (32.1) | <0.001 | 2,473  (29.6) | 2,476  (29.6) | 0.959 | 0.001 |
| **Aspirin** | 4,127  (53.4) | 13,482  (43.0) | <0.001 | 3,689  (44.1) | 3,663  (43.8) | 0.685 | 0.006 |
| **Clopidogrel** | 1,050  (10.9) | 4,600  18.2) | <0.001 | 994  (11.9) | 972  (11.6) | 0.597 | 0.008 |
| **Diuretics** | 5,137  (53.6) | 16,851  (66.7) | <0.001 | 4,657  (55.7) | 4,700  (56.2) | 0.503 | 0.010 |
| **Finerenone** | 10  (0.1) | 13  (0.1) | 0.087 | 10  (0.1) | 10  (0.1) | 1 | <0.001 |
| **Eplerenone** | 53  (0.6) | 200  (0.8) | 0.019 | 51  (0.6) | 62  (0.7) | 0.299 | 0.016 |
| **Spironolactone** | 883  (9.2) | 3,176  (12.6) | <0.001 | 838  (10.0) | 851  (10.2) | 0.739 | 0.005 |
| **LABORATORY** | | | | | | | |
| **eGFR***  **Mean ± SD** | 65.3  ± 31.7 | 37.1  ± 29.3 | <0.001 | 62.5  ± 31.3 | 52.6  ± 32.3 | <0.001 | 0.312 |
| **eGFR categories (ml/min/1.73m^2^)** | | | | | | | |
| **>90** | 4,552  (47.5) | 6,284  (24.9) | <0.001 | 3,532  (42.2) | 3,533  (42.2) | 0.988 | <0.001 |
| **60-89** | 6,431  (67.1) | 11,690  (46.3) | <0.001 | 5,316  (63.6) | 5,298  (63.3) | 0.773 | 0.004 |
| **30-59** | 5,416  (56.5) | 15,460  (61.2) | <0.001 | 4,859  (58.1) | 4,994  (59.7) | 0.034 | 0.033 |
| **15-29** | 2,275  (23.7) | 12,375  (49.0) | <0.001 | 2,236  (26.7) | 2,290  (27.4) | 0.347 | 0.015 |
| **< 15** | 1,261  (13.1) | 9,033  (35.8) | <0.001 | 1,251  (15.0) | 1,278  (15.3) | 0.560 | 0.009 |
| **Proteinuria (Microalbumin mg/g)** | | | | | | | |
| **0 - 30** | 1,246  (13.0) | 2,055  (8.1) | <0.001 | 996  (11.9) | 1,031  (12.3) | 0.407 | 0.013 |
| **30 - 300** | 1,332  (13.9) | 3,092  (12.2) | <0.001 | 1,147  (13.7) | 1,204  (14.4) | 0.205 | 0.020 |
| **>300** | 837  (8.7) | 3,081  (12.2) | <0.001 | 778  (9.3) | 797  (9.5) | 0.615 | 0.008 |
| **Cholesterol mg/dL (Mean ± SD)** | 178.7  ± 58.1 | 163.1  ± 56.7 | <0.001 | 175.5  ± 56.4 | 174.6  ± 62.9 | 0.425 | 0.015 |

Table showing the survival analysis for NT-proBNP all cause GN and primary GN sub-type cohort. Survival analysis estimates the probability of an outcome at a respective time over 5 years follow-up from the index event. Statistical analysis was performed using the' Analytics' functionality on TriNetX which used the R Survival package v3.2-3 for its analysis. Results are reported as hazard ratio (HR),95% Confidence interval (CI) and log-rank tests(χ^2^). A p-value <0.05 was accepted as the level of statistical significance. *Estimated glomerular filtration rate ml/min/1.73m2 (MDRD formula)

**Appendix Table 7.** Sensitivity survival analysis adjusted for CKD; Number of patients with each MACE outcome, Hazard ratio and 95% confidence interval for Troponin I at a threshold of 18ng/L.

|  | **Outcome** | **Troponin I** | | | | | | | |
| --- | --- | --- | --- | --- | --- | --- | --- | --- | --- |
|  |  | **<18 ng/L** | | **≥18 ng/L** | | | | | |
| **All Cause GN** |  | **Cohort**  **N=** | **Outcome**  **N=** | **Cohort**  **N=** | **Outcome**  **N=** | **HR** | **95% CI** | **χ^2^** | **P-Value** |
|  | **MACE** | 7,516 | 2,880 | 5,356 | 3,016 | 1.76 | (1.67, 1.86) | 66.619 | <0.0001 |
|  | **IHD** | 10,784 | 2,276 | 9,152 | 2,668 | 1.56 | (1.48, 1.65) | 49.737 | <0.0001 |
|  | **Angina** | 15,252 | 765 | 15,292 | 985 | 1.35 | (1.23, 1.48) | 20.045 | <0.0001 |
|  | **Acute MI** | 15,544 | 1,332 | 13,827 | 1,955 | 1.76 | (1.65, 1.89) | 46.217 | <0.0001 |
|  | **Heart Failure** | 11,818 | 2,142 | 10,300 | 2,936 | 1.77 | (1.67, 1.87) | 35.896 | <0.0001 |
|  | **Atrial fibrillation and flutter** | 14,210 | 1,299 | 13,577 | 1,696 | 1.44 | (1.34, 1.54) | 7.086 | 0.008 |
|  | **Cerebral Infarction** | 15,256 | 922 | 15,257 | 1,115 | 1.25 | (1.15, 1.37) | 10.267 | 0.001 |
|  | **Deceased** | 16,780 | 3,812 | 16,664 | 5,363 | 1.48 | (1.42, 1.54) | 59.303 | <0.0001 |
| *Table showing the survival analysis for Troponin I in all cause GN sub-group adjusted for CKD. Survival analysis estimates the probability of an outcome at a respective time over 5 years follow-up from the index event. Statistical analysis was performed using the' Analytics' functionality on TriNetX which used the R Survival package v3.2-3 for its analysis. Results are reported as hazard ratio (HR),95% Confidence interval (CI) and log-rank tests(χ2). A p-value <0.05 was accepted as the level of statistical significance.* | | | | | | | | | |

| **Appendix Table 8.** Sub-group survival analysis adjusted for CKD; Number of patients with each MACE outcome, Hazard ratio and 95% confidence interval for NTproBNP at a threshold of 400 pg/ml. | | | | | | | | | |
| --- | --- | --- | --- | --- | --- | --- | --- | --- | --- |
|  | **Outcome** | **NTproBNP** | | | | | | | |
|  |  | **<400 pg/ml** | | **≥400 pg/ml** | | | | | |
| **All Cause GN** |  | **Cohort**  **N=** | **Outcome**  **N=** | **Cohort**  **N=** | **Outcome**  **N=** | **HR** | **95% CI** | **χ^2^** | **P-Value** |
|  | **MACE** | 3,539 | 1,235 | 2,794 | 1,500 | 1.99 | (1.85, 2.15) | 32.823 | <0.0001 |
|  | **IHD** | 5,297 | 862 | 4,984 | 1,213 | 1.78 | (1.63, 1.94) | 12.490 | <0.0001 |
|  | **Angina** | 7,324 | 336 | 7,533 | 407 | 1.31 | (1.13, 1.51) | 0.092 | 0.761 |
|  | **Acute MI** | 7,449 | 478 | 7,087 | 750 | 1.90 | (1.67, 2.13) | 22.393 | <0.0001 |
|  | **Heart Failure** | 5,685 | 906 | 4,871 | 1,441 | 2.32 | (2.14, 2.52) | 70.087 | <0.0001 |
|  | **Atrial fibrillation and flutter** | 7,453 | 535 | 6,512 | 794 | 1.93 | (1.73, 2.16) | 19.859 | <0.0001 |
|  | **Cerebral Infarction** | 7,549 | 341 | 7,501 | 439 | 1.45 | (1.26, 1.67) | 12.442 | <0.0001 |
|  | **Deceased** | 8,255 | 1,255 | 8,231 | 2,638 | 2.41 | (2.25, 2.57) | 30.110 | <0.0001 |
| *Table showing the survival analysis for NTproBNP in all cause GN sub-group adjusted for CKD. Survival analysis estimates the probability of an outcome at a respective time over 5 years follow-up from the index event. Statistical analysis was performed using the' Analytics' functionality on TriNetX which used the R Survival package v3.2-3 for its analysis. Results are reported as hazard ratio (HR),95% Confidence interval (CI) and log-rank tests(χ2). A p-value <0.05 was accepted as the level of statistical significance.* | | | | | | | | | |
